# Quantifying Inorganic Nitrogen Assimilation by *Synechococcus* Using Bulk and Single-Cell Mass Spectrometry: A Comparative Study

**DOI:** 10.3389/fmicb.2018.02847

**Published:** 2018-11-27

**Authors:** Marco Giardina, Soshan Cheong, Christopher E. Marjo, Peta L. Clode, Paul Guagliardo, Russell Pickford, Mathieu Pernice, Justin R. Seymour, Jean-Baptiste Raina

**Affiliations:** ^1^Climate Change Cluster, University of Technology Sydney, Sydney, NSW, Australia; ^2^Mark Wainwright Analytical Centre, University of New South Wales, Kensington, NSW, Australia; ^3^Centre for Microscopy Characterisation and Analysis, The University of Western Australia, Perth, WA, Australia; ^4^UWA School of Biological Sciences, The University of Western Australia, Perth, WA, Australia; ^5^UWA Oceans Institute, The University of Western Australia, Perth, WA, Australia; ^6^Bioanalytical Mass Spectrometry Facility, University of New South Wales, Sydney, NSW, Australia

**Keywords:** *Synechococcus*, nitrogen, EA-IRMS, NanoSIMS, ToF-SIMS, metabolic heterogeneity, single cell

## Abstract

Microorganisms drive most of the major biogeochemical cycles in the ocean, but the rates at which individual species assimilate and transform key elements is generally poorly quantified. One of these important elements is nitrogen, with its availability limiting primary production across a large proportion of the ocean. Nitrogen uptake by marine microbes is typically quantified using bulk-scale approaches, such as Elemental Analyzer-Isotope Ratio Mass Spectrometry (EA-IRMS), which averages uptake over entire communities, masking microbial heterogeneity. However, more recent techniques, such as secondary ion mass spectrometry (SIMS), allow for elucidation of assimilation rates at the scale at which they occur: the single-cell level. Here, we combine and compare the application of bulk (EA-IRMS) and single-cell approaches (NanoSIMS and Time-of-Flight-SIMS) for quantifying the assimilation of inorganic nitrogen by the ubiquitous marine primary producer *Synechococcus*. We aimed to contrast the advantages and disadvantages of these techniques and showcase their complementarity. Our results show that the average assimilation of ^15^N by *Synechococcus* differed based on the technique used: values derived from EA-IRMS were consistently higher than those derived from SIMS, likely due to a combination of previously reported systematic depletion as well as differences in sample preparation. However, single-cell approaches offered additional layers of information, whereby NanoSIMS allowed for the quantification of the metabolic heterogeneity among individual cells and ToF-SIMS enabled identification of nitrogen assimilation into peptides. We suggest that this coupling of stable isotope-based approaches has great potential to elucidate the metabolic capacity and heterogeneity of microbial cells in natural environments.

## Introduction

Stable isotopes have been used extensively in microbial ecology (Boschker and Middelburg, 2002) to quantify microbial metabolic activities (Dumont and Murrell, 2005), determine the uptake rate of specific molecules (Pelz et al., 1998; Bronk, 1999), track nutrient transfer between different organisms (Van Den Meersche et al., 2004, 2011; Raina et al., 2017), and monitor chemical transformations through a range of biotic or abiotic processes (Matwiyoff and Ott, 1973; Post, 2002). In microbial oceanography, the assimilation rate of inorganic nitrogen by marine micro-organisms is traditionally quantified using large sample volumes (Garside, 1981; Mccarthy et al., 1992), whereby molecules labeled with the rare stable isotope ^15^N are often employed as a tracer used to enrich liters of seawater, during incubation periods ranging from hours to days (Evrard et al., 2010; Van Den Meersche et al., 2011). The microbial biomass is then concentrated (by filtration or centrifugation) before measuring the ^15^N/^14^N ratio of the entire community with mass spectrometry.

One of the most widely used techniques to measure stable isotope ratios is elemental analyzer-isotope ratio mass spectrometry (EA-IRMS) (Boschker and Middelburg, 2002; Muccio and Jackson, 2009). EA-IRMS has been widely applied for studying microbially mediated biogeochemical cycles (Montoya et al., 1996; Boschker et al., 1998; Hinrichs et al., 1999) and has become an important tool for tracking nutrient transfer among microbes (Pel et al., 2003). However, bulk-scale approaches such as EA-IRMS are, by definition, disconnected from the metabolic activities of the individual microscopic organisms targeted, averaging out their metabolic activities. Single-cell approaches, on the other hand, offer the potential to unveil the metabolic and phenotypic diversity present in microbial communities and to more accurately quantify how their activities might scale-up to affect oceanic processes.

The application of imaging mass spectrometry, such as nano-scale secondary ion mass spectrometry (NanoSIMS) and Time of Flight-SIMS (ToF-SIMS), enables direct investigation of the metabolic activities and functions of specific micro-organisms within complex microbial communities at the single-cell level (Wagner, 2009; Watrous and Dorrestein, 2011; Gao et al., 2015). The power of these instruments relies on their capacity to visualize and quantify ions at micro-scale resolution. Both NanoSIMS and ToF-SIMS use a high energy primary ion beam to blast the sample surface (Wagner, 2009; Gao et al., 2015), ejecting secondary ions derived from the sample in a process called sputtering (Watrous and Dorrestein, 2011). The secondary ions produced are subsequently directed into a mass spectrometer then separated by different mass-to-charge ratios using an electrostatic field (NanoSIMS) or time-of-flight tube (ToF-SIMS) (Kilburn and Clode, 2014). While both ToF-SIMS and NanoSIMS employ a high energy primary ion beam (Passarelli and Winograd, 2011; Kilburn and Clode, 2014), they differ in their currents and beam diameters, which has significance for determining the spatial resolution of the instruments – the smaller the beam the higher the resolution. The primary ion beam in ToF-SIMS can be narrowed down to the sub-micron scale providing a spatial resolution between 1 to 5 μm. The comparatively gentler sputtering of this instrument, relative to NanoSIMS, leads to the production of fewer secondary ions, impacting the accuracy of quantitative analyses, but generating intact molecular ions and allowing their identification (Watrous and Dorrestein, 2011; Hoefler and Straight, 2014; Kilburn and Clode, 2014). In contrast, the smaller primary ion beam of the NanoSIMS enables a spatial resolution of analysis close to 50 nm, while the more intense sputtering of this instrument generates higher secondary ion flux allowing for very accurate elemental quantification but destroying the chemical structure of the molecules (Watrous and Dorrestein, 2011; Kilburn and Clode, 2014).

NanoSIMS has been used extensively in microbial ecology since the early 2000s to quantify nutrient uptake rates by individual cells (Alonso et al., 2012; Foster et al., 2013; Krupke et al., 2013), characterize bacterial metabolisms (Finzi-Hart et al., 2009; Musat et al., 2012; Terrado et al., 2017) and study microbe-microbe (Foster et al., 2011; Thompson et al., 2012; Bonnet et al., 2016; Arandia-Gorostidi et al., 2017; Raina et al., 2017; Carpenter et al., 2018; Samo et al., 2018) and animal-microbe (Lechene et al., 2006, 2007; Pernice et al., 2012; Rädecker et al., 2018) interactions at the single-cell level (Musat et al., 2016). ToF-SIMS has been used to characterize the spatial distribution of microbial biomarkers (Thiel et al., 2007) and detect the distribution of intact molecules in cells (Vaidyanathan et al., 2008). Recently, ToF-SIMS and NanoSIMS were coupled to quantify the transfer of organosulfur molecules between microalgae and bacteria (Raina et al., 2017) and nutrient transfer between fungi and bacteria (Worrich et al., 2017).

Here we combine EA-IRMS, NanoSIMS, and ToF-SIMS to quantify nitrogen uptake and metabolism by the ubiquitous marine cyanobacteria *Synechococcus* from the bulk-scale down to the scale of individual cells. While there is extensive literature on nitrogen requirements of this important microorganism at the bulk scale (Glibert and Ray, 1990; Bronk, 1999; Moore et al., 2002), little is known about these dynamics at the scale of individual cells. By using three different stable isotope analytical techniques, we aim to contrast their specific advantages and disadvantages and highlight their complementarity when studying small bacterial cells.

## Materials and Methods

### *Synechococcus* Culture Maintenance

*Synechococcus* sp. CS-94 RRIMP N1 (S1) was cultured for 7 days in a modified form of f/2 (-Si) medium that combines the nutrients of f/2 medium (Guillard, 1975) and the artificial salt solutions of the Enriched Seawater Artificial Water (ESAW) (Berges et al., 2001), with the latter used instead of natural filtered seawater, which can contain biologically available nitrogen. The culture was maintained under conditions mirroring the natural environmental preferences of this organism: e.g., temperature of 23°C and illumination with an incident photon irradiance of ∼180 μmol photons m^-2^ s^-1^ (12 h: 12 h light: dark cycle).

### Experimental Design and Samples Collection

On the day of the experiment, 50 ml of *Synechococcus* culture was centrifuged at 1,500 *g* for 15 min. To quantify the assimilation of nitrogen by the *Synechococcus* cells, the supernatant was removed and replaced with fresh growth media containing ^15^N-labeled Sodium Nitrate (NaNO_3_; final concentration 8.82 × 10^-4^M; ^15^N, 98%+, Cambridge Isotopes Laboratories, Inc., Cambridge, MA, United States), as the sole source of biologically available nitrogen. A *Synechococcus* culture subsample was centrifuged, as described above, but resuspended in fresh growth medium containing natural abundance of ^15^N and acted as an unlabelled control. A 6-h incubation was performed with 1.5 ml samples collected sequentially through time at the following time-points: 0 min (T_0_; sample not exposed to ^15^N enrichment, see above), 15 min (T_1_), 30 min (T_2_), 1 h (T_3_), 2 h (T_4_), 4 h (T_5_), 6 h (T_6_). Samples were collected in triplicate for IRMS analysis and in duplicate for ToF-SIMS and NanoSIMS. All samples were centrifuged for 15 min at 1,500 *g*, the supernatant was removed and replaced with 500 μl of paraformaldehyde (1% final concentration) diluted in buffer (0.1 M Sucrose, 1 × PBS). Samples were fixed for 24 h at 4°C, washed three times with buffer (0.1 M Sucrose in 1 × PBS) in order to eliminate any residual paraformaldehyde before being prepared for specific instruments.

### EA-IRMS: Sample Preparation and Analysis

Cells were collected by centrifuging the samples at 1,500 *g* for 15 min, the buffer was removed and the pellet dried in the oven at 60°C for 48 h. Glutamic acid was used as a standard, whereby different ratios of ^15^N-labeled glutamic acid (^15^N-Glu) (L-Glutamic acid, 98 atom% ^15^N, Sigma-Aldrich) and non-labeled glutamic acid (^nat^N-Glu) (L-Glutamic acid, ≥99% HPLC-grade, Sigma-Aldrich) were prepared at a final concentration 1 mg ml^-1^: 100% ^nat^N-Glu, 1:50,000 (^15^N-Glu:^nat^N-Glu), 1:10,000, 1:5,000, 1:1,000, 1:500, 1:100, 1:50, 1:10. The different standard solutions were reduced to dryness in an oven at 60°C for 48 h. Approximately 0.250 mg of either samples or standards were weighed into tin capsules and loaded into the autosampler of the EA-IRMS.

Samples were analyzed on a Thermo Fisher Scientific EA-IRMS system featuring Flash 2000 organic elemental analyzer and Delta Plus IRMS coupled via an EA-Isolink. The carbon and nitrogen in the sample was converted to CO_2_ and N_2_ within the elemental analyzer before transfer to the mass spectrometer for isotope ratio analysis. Values obtained were corrected using the Vienna Pee Dee Belemnite (VPDB) standard. Instrument operation was performed using IsoDat software (Thermo Fisher Scientific, Waltham, MA, United States). To ensure the accuracy of the EA-IRMS, we measured the dilution series of glutamic acid standards (i.e., increasing ratios of ^15^N/^14^N). These standards were used as the reference to quantify the assimilation of ^15^N by the *Synechococcus* culture (Supplementary Figure 1). The measured ^15^N atom fraction in the ^nat^N-Glu standard was 0.36%, and the value measured in the highest dilution, 1:10 (^15^N:^14^N), was 9.21% (Supplementary Figure 1).

### Sample Preparation for SIMS

In order to remove the sucrose buffer and obtain a monolayer of cells, the samples were diluted ten times in sterile, filtered Milli-Q water (0.22 μm pore size, Minisart syringe filters, Sartorius, Göttingen, Germany) and 50 μl were immediately placed onto silicon wafers (7.07 mm × 7.07 mm, Type P, diameter: 4 inches, orientation: <111>, ProSciTech, Townsville, QLD, Australia), dried at 45°C and stored inside a desiccator, protected from light until SIMS analysis.

### NanoSIMS Analysis

We used the NanoSIMS 50 (Cameca, Gennevilliers, France) at the Centre for Microscopy, Characterisation and Analysis (CMCA) at The University of Western Australia. This instrument allows for simultaneous collection of up to five isotopic species (here: ^12^C_2_^-^, ^12^C^13^C^-^, ^12^C^14^N^-^, ^12^C^15^N^-^, and ^32^S). Enrichment of the rare isotope ^15^N was confirmed by an increase in the ^15^N/^14^N ratio above the natural abundance value recorded in controls (equal to 0.374% ± 0.001 for nitrogen). Different pre-sputtering lengths and current intensities were tested. The analysis was performed as followed: samples were pre-sputtered for 3.5 min at 500 pA Cs^+^ beam (D1 = 1) on 30 μm^2^ areas (256 × 256 pixel), followed by automatic horizontal and vertical secondary ion beam centering. We selected the above conditions because counts did not increase with longer sputtering, we therefore assumed that the beam reached the inner part of the cells. The analysis was then performed by rastering a 2 pA beam (D1 = 2) over 25 μm^2^ areas (256 × 256 pixels); three planes were recorded per area with a dwell time of 3 ms per pixel. The instrument was operated with a high mass resolving power (in the range of 9,000), allowing the separation of isobaric interferences. Images were analyzed using the Fiji software package^1^ (Schindelin et al., 2012) combined with the Open-MIMS plug-in^2^. All images were dead-time corrected (Hillion et al., 2008); the individual planes were then summed prior to extracting counts from the images. Isotopic quantification data were extracted from the mass images by manually drawing regions of interest around each single bacterial cell using the ^12^C^14^N^-^ image as mask. The raw NanoSIMS data are shown in Supplementary Table 3.

### ToF-SIMS Analysis

ToF-SIMS is a surface analysis technique where only the uppermost molecular layers are analyzed. The data collected by ToF-SIMS can be visualized as both (i) an accumulated mass spectrum from the bulk surface, and (ii) a two-dimensional image showing the intensity distribution of the specific secondary ions in the area analyzed (Fearn, 2015). In order to probe the ^14^N and ^15^N species within the cells, and not only on the cell surfaces, depth profiling analysis was performed, where the cells were sputtered through in a dual beam configuration with alternating analysis and sputtering cycles. Using this approach, the resultant spectrum in images is a composite of all spectra collected throughout the depth profile and is more comparable to data obtained using NanoSIMS that also sputters through the sample. ToF-SIMS analysis was conducted using the TOF.SIMS 5 instrument (ION-TOF GmbH, Münster, Germany) at the Mark Wainwright Analytical Centre (MWAC), University of New South Wales. This instrument is equipped with a bismuth liquid metal cluster ion gun for analysis, an argon gas cluster ion gun for depth profile sputtering, and an electron flood gun for charge compensation. The “non-interlaced” mode was employed, with each cycle consisting of 1 scan of sputtering (∼1.5 s), followed by 2 scans of data acquisition, and a 0.5 s pause in between the analyses. Sputtering was performed using a 10 keV Ar_2000_^+^ cluster ion beam rastering over a 340 × 340 μm^2^ area. Mass spectral data for the images was acquired using a 30 keV Bi_3_^+^ ion beam, analyzing a 100 × 100 μm^2^ square in the central region. The analysis beam was operated in the “spectrometry” mode that compromises the lateral spatial resolution of the image but maximizes the mass resolution (m/Δm > 5000). All analyses were conducted in the negative polarity. Spectra were mass calibrated using the masses of C^-^, C_2_^-^, C_3_^-^, C_4_^-^, and C_5_^-^ molecules. Prior to each depth profiling analysis, the analysis area was first identified by surveying the ^12^C^14^N^-^ (m/z 26) maps acquired in the “fast-imaging” mode for high-lateral-resolution (∼195 nm) images. During this initial imaging analysis, no more than two scans were acquired for each area of analysis to ensure the ion dose density was kept below the static SIMS limit that ensures minimal damage to the sample surface. Data processing and evaluation was conducted using the SurfaceLab six software package (ION-TOF GmbH, Münster, Germany). The raw ToF-SIMS data are shown in Supplementary Table 4.

### Peak Deconvolution Following ToF-SIMS Analysis

Incorporation of ^15^N into a biological system using imaging ToF-SIMS is best studied using the ^12^C^15^N (m/z 27.000109) and ^12^C^14^N (m/z 26.0003074) anions that can be subsequently used to calculate the ^12^C^15^N/^12^C^14^N ratio. However, ^12^C^15^N has a significant overlap with ^13^C^14^N (m/z 27.006429) and a nearby peak at ^12^C_2_^1^H_3_ (m/z 27.023475). The mass spectral resolution of ToF-SIMS is less than NanoSIMS, so a deconvolution of the data is required to extract the true peak intensities around m/z 27. In addition to the peak overlap, the peaks in ToF-SIMS mass spectra are asymmetric, exhibiting a tail toward higher mass, so a Gaussian-Lorentzian function incorporating a tailing term was used to fit the data correctly (Beamson and Briggs, 1992). For multi-peak fitting, the tailing term and peak width were fixed for each peak, with only position and height varying for individual peaks. Fitting was achieved by minimizing Chi^2^ against the experimental data. The ^12^C^14^N peak at m/z 26 is substantially more intense than the ^12^C^15^N peak at m/z 27, leading to poor fits in the latter region unless each region is fitted separately. The m/z 26 region was fitted using a single peak for ^12^C^14^N, while the region at m/z 27 was fitted with two peaks representing ^12^C^15^N, ^13^C^14^N (the peak at ^12^C_2_^1^H_3_ was sufficiently well resolved and did not require fitting; Supplementary Figure 2). A batch fitting process was performed on the average spectrum within each ROI and implemented by the authors in Python 3.1 using the curve_fit function found in the Scipy Optimize module. Peak fitting of the ^12^C^14^N peak was readily performed in a single step using this code. In the case of the 3-peak fitting of the m/z 27 region, each fit was evaluated graphically by eye, and poor fits were reprocessed individually using different start values for position and height to ensure all peaks appeared in the expected positions based on their theoretical m/z values. An equivalent process was adopted for calculation of the higher mass molecular ion ratios of ^12^C_3_^15^N/^12^C_3_^14^N and ^12^C^15^N^16^O/^12^C^14^N^16^O. However, the mass resolution of the ToF-SIMS was not sufficient to enable deconvolution of the ^13^C-containing interferences (^13^C_3_^14^N and ^13^C^14^N^16^O), resulting in anomalously high peak fitting ratios (approximately 4 atom percent higher than natural abundance in T_0_–T_3_). Nevertheless, the full time-series data clearly showed a positive trend confirming the increasing incorporation of ^15^N in these molecular ions.

### ^15^N Atom Fraction Calculation

The measured isotope ratios were converted to ^15^N atom fraction (Atom%), which gives the percentage of a specific atom within the total number of atoms. In this case, we calculated the percentage of ^15^N within the total number of nitrogen atoms (^15^N + ^14^N) following the formula:

$$Atom\%=\frac{{}^{15}\text{N}}{{}^{15}\text{N}{+}^{\text{14}}\text{N}}\times 100$$

### Statistical Analysis

All statistical analyses were carried out using SPSS (version 23; IBM Corporation, Armonk, NY, United States). Data were first tested for normality and homogeneity of variance using Shapiro-Wilk and Levene’s tests, respectively. When the data was not normally distributed and/or the variances were not homogeneous, comparisons of ^15^N enrichment were carried out using the Mann–Whitney *U*-test. Pairwise comparisons between each method, at each time point, were conducted using Kruskal-Wallis *H*-test with Dunn’s *post hoc* tests and Bonferroni adjustment. A summary of the statistical results is reported in Supplementary Tables 1, 2.

## Results and Discussions

EA-IRMS, NanoSIMS, and ToF-SIMS were used to quantify ^15^NO_3_^-^ assimilation by *Synechococcus* at both the bulk and single-cell level. Correlations between EA-IRMS and NanoSIMS approaches have previously been reported, whereby these two techniques have been combined to measure the metabolic activities of multiple marine microorganisms, including the mixotrophic alga *Ochromonas* spp. BG-1 (Terrado et al., 2017), a subseafloor chemoautotrophic member of the *Campylobacter* (Mcnichol et al., 2018) and several nitrogen-fixing cyanobacteria (Popa et al., 2007; Ploug et al., 2010; Foster et al., 2013; Krupke et al., 2013). However, this study is the first to additionally compare the use of EA-IRMS and NanoSIMS with ToF-SIMS, which can provide further insight on the fate of nitrogen within *Synechococcus* cells.

**FIGURE 1:**
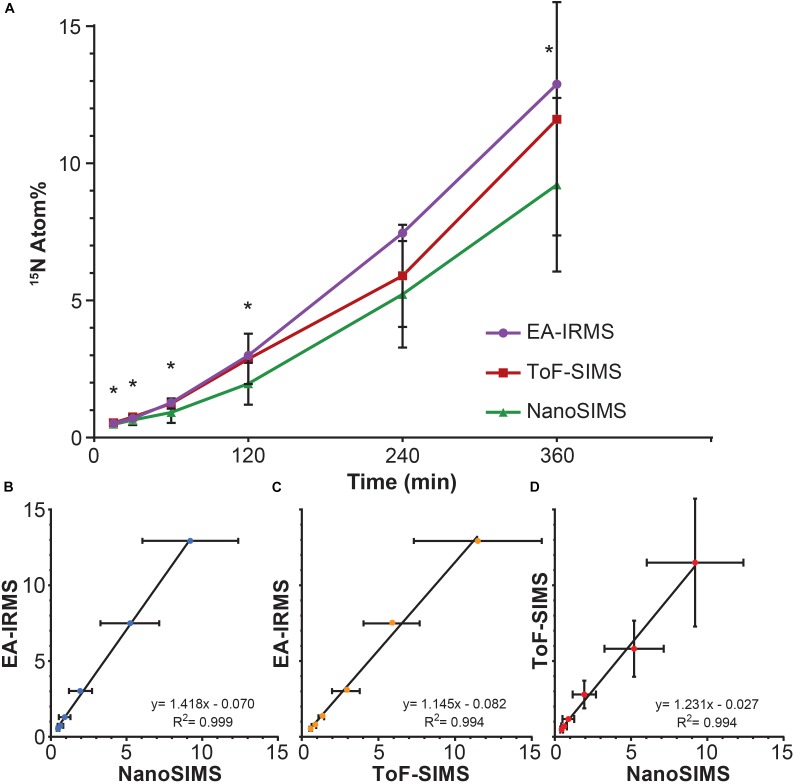
^15^N assimilation by *Synechococcus* sp. between **(A)** EA-IRMS (purple), ToF-SIMS (red), and NanoSIMS (green). Asterisk denote significant differences between ToF-SIMS and NanoSIMS (see Supplementary Table 1). Relationship between different ^15^N measurement (in Atom%) performed with **(B)** EA-IRMS and NanoSIMS, **(C)** EA-IRMS and ToF-SIMS and **(D)** NanoSIMS and ToF-SIMS. All slopes differed significantly from 0 (ANOVA, *p* < 0.05). All measurements were carried out on different samples collected from the same culture flask (*n* = 1 biological replicate). Error bars: standard deviation of three technical replicates measured with EA-IRMS (technical replicates) and single cells measured with NanoSIMS and ToF-SIMS. For number of replicates refer to Supplementary Table 2.

All three techniques revealed that the *Synechococcus* cells became significantly enriched in ^15^N after only 15 min of incubation (T_0_ vs. T_1_: EA-IRMS *t*-test *p* = 0.000; ToF-SIMS *p* = 0.002; NanoSIMS *p* = 0.000; Supplementary Table 2). The ^15^N atom fraction increased over the 6 h incubation period, from 0.371% ± 0.003 (T_0_) to 12.89% ± 0.06 (T_6_) for EA-IRMS; 0.374% ± 0.007 to 9.2% ± 3.2 for NanoSIMS; and 0.46% ± 0.08 to 11.6% ± 4.2 for ToF-SIMS (Figure 1A). However, the three instruments exhibited substantial discrepancies (Figure 1A and Supplementary Table 1): after 6 h of incubation, the enrichment values quantified by EA-IRMS were on average 10% higher than those recorded by ToF-SIMS, and 28% higher than those from NanoSIMS (Supplementary Table 1). Previous studies have also reported imperfect matches between EA-IRMS and NanoSIMS (Kopf et al., 2015; Terrado et al., 2017), which have been ascribed to a combination of differences in sample preparations together with known systematic depletion (by 1–10% of the heaviest isotope) due to systematic fractionation during SIMS analysis (Fitzsimons et al., 2000). In addition to fractionation effect, the differences between instruments observed here are most likely due to two factors: (i) although all samples were prepared using the same protocol, SIMS samples had to be subsequently diluted 10-fold directly prior to drying in order to obtain an even layer of cells and avoid their superimposition. Because this dilution was the only difference between EA-IRMS and the two SIMS techniques, it is likely that this step triggered a loss of water-soluble nitrogen compounds from the cells. In addition, (ii) the mass resolving power of the two SIMS instrument is not equal. This point is best exemplified by the artefactual enrichment of the ToF-SIMS samples at T_0_ and is due to a combination of peak overlap (differences between isobaric peaks such as ^11^B^16^O^-^ and ^12^C^15^N^-^ cannot be resolved) and peak asymmetry (exhibiting a tail toward higher mass). This latter point likely explains why the ToF-SIMS-derived enrichments are higher on average than the NanoSIMS ones (Supplementary Table 1). Despite variations between techniques, linear regression analyses showed a strong positive relationship between measurements from the three instruments (Figures 1B–D). This indicates that the measured increase in ^15^N enrichment through time was consistent across all three techniques.

**FIGURE 2:**
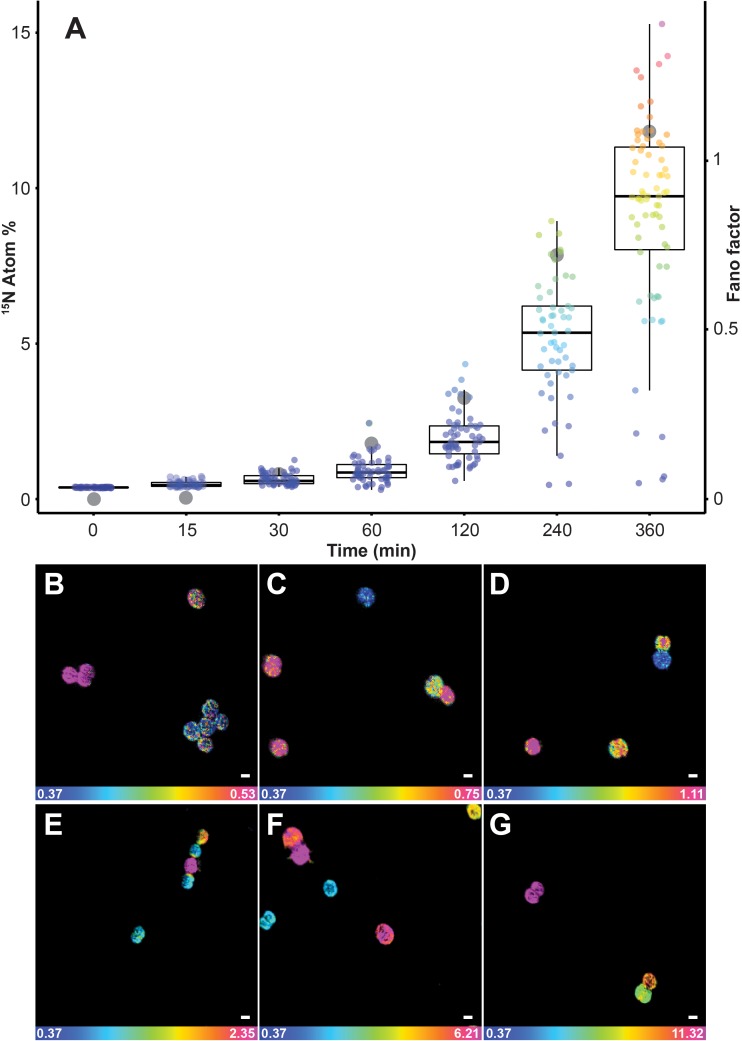
Quantification of ^15^N uptake by *Synechococcus* cells through time at single-cell level using NanoSIMS. **(A)** Box plot showing an increase in single-cell heterogeneity (lower and upper hinges correspond to the 25th and 75th percentiles) as well as the Fano factor (ratio of sample variance to sample mean; indicated in the figure by gray dots) which measures the heterogeneity of the ^15^N assimilation. Representative NanoSIMS images showing the distribution of ^15^N/^14^N ratio after **(B)** 15 min, **(C)** 30 min, **(D)** 1 h, **(E)** 2 h, **(F)** 4 h, and **(G)** 6 h. Scale bar: 1 μm. Note: the scale of the images increase from **(B–G)** to highlight the cellular heterogeneity: blue represent natural ^15^N atom fraction and magenta represent the third quartile of each respective data point. For number of analyzed cells refer to Supplementary Table 2. All measurements were carried out on a sample taken from the same culture flask (*n* = 1 biological replicate).

^15^N enrichments recorded using SIMS were considerably more variable than the values reported with EA-IRMS. Although bulk measurements with EA-IRMS provided an accurate quantification of the metabolic activities in the whole *Synechococcus* culture, it inherently masked the disparities in nitrogen enrichment at the single-cell level. The averaged nitrogen ratio recorded through NanoSIMS indicated significant ^15^N uptake after 15 min of incubation (0.48% ± 0.09), but this technique also allowed us to identify a high variability in ^15^N enrichment between single cells (Figure 2A). Up to an hour after the start of the ^15^N exposure, approximately 7% of the cells were not enriched, while up to 5% of the cells were more than twice as enriched as the population average. In comparison, after 4 h, even the cells that were the least enriched exhibited ^15^N levels that were twice as high as the natural abundance. The heterogeneity in ^15^N content between cells, in the 50 ml culture flask we investigated, became more pronounced in the later time points, which was quantified using two different measures of the dispersion of observations: the interquartile range (IQR) and the Fano factor (Figure 2). The IQR increased from 0.9 after 2 h to 3.3 after 6 h of incubation, similarly the Fano factor increased from 0.01 after 15 min to 1.08 after 6 h. This variability in enrichment among cells exposed to the same conditions is time-dependent but its underlying causes can result from several biological and methodological factors which have previously been identified (Musat et al., 2008; Finzi-Hart et al., 2009; Ploug et al., 2010; Woebken et al., 2012).

Among the biological factors impacting cell-to-cell variation in NanoSIMS-derived ^15^N enrichment, heterogeneity in the assimilation rate is likely to play an important role. Indeed, microbial populations are composed of a collective of individual cells, each potentially displaying different metabolic activity (Adams, 2000; Johnson et al., 2012) and behavioral traits (Crespi, 2001). In some cases, the differences between cells are not due to genetic diversity, but are the result of phenotypic heterogeneity, which is based on the stochasticity of several molecular mechanisms that induce differences between single cells, even in the absence of genetic and environmental variation (Ackermann, 2015). For example, unequal cell division may lead to a different distribution of key components, such as enzymes, ribosomes, or pigments, inducing significant physiological differences in the daughter cells (Huh and Paulsson, 2011). Heterogeneity in ^15^N-enrichment observed in NanoSIMS can also be due to differences in cells life cycle, in cell sizes (Lidstrom and Konopka, 2010) or in metabolic rates, which is consistent with observations among other unicellular cyanobacteria (Foster et al., 2013). This heterogeneity within a cell population might deliver ecological benefits, including the division of labor between individuals and the survival of specific phenotypes in fluctuating environments (Lidstrom and Konopka, 2010; Ackermann, 2015; Schreiber et al., 2016). Within natural marine ecosystems, where the distribution of nutrients is highly patchy at the microscale (Stocker, 2012), such inter-cellular variability in metabolism is highly likely to be widespread. In addition, a range of methodological factors can also affect cell-to-cell heterogeneity in enrichment measured by NanoSIMS including the orientation of the cells, their biovolume and elemental density (Musat et al., 2014, 2016; Pernice et al., 2015; Achlatis et al., 2018).

**FIGURE 3:**
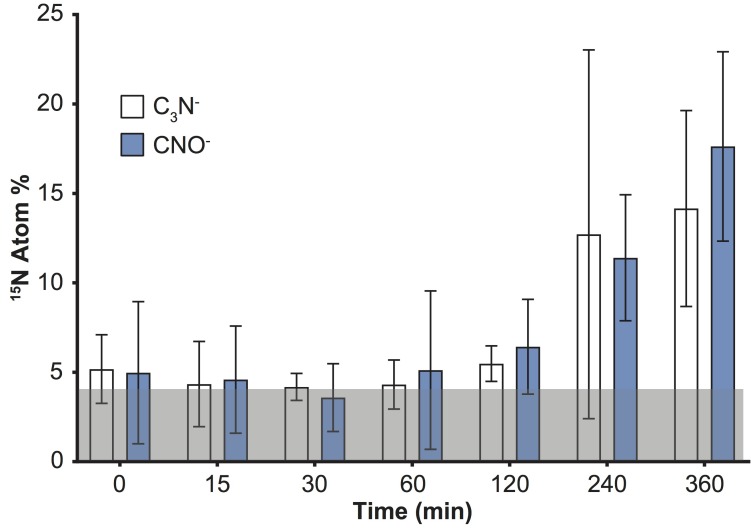
Detection of ^15^N incorporation into peptides by quantifying C_3_N^-^ and CNO^-^ in *Synechococcus* cells with ToF-SIMS. Note: deconvolution of neighboring peaks (^13^C^12^C_2_^14^N and ^13^C^14^N^16^O), resulted in erroneously offset values (by approximately 4 Atom% throughout the time series; shaded gray area). Error bars: standard deviation of single cell measurements. For number of replicates refer to Supplementary Table 2. All measurements were carried out on a sample taken from the same culture flask (*n* = 1 biological replicate).

An increase in the variability of ^15^N enrichment among single cells/aggregates was also recorded over time using ToF-SIMS (Figure 1), and became more pronounced in the later time-points (IQR: 2 h = 1.3; 4 h = 1.7; 6 h = 4.7). We subsequently investigated the ^15^N incorporation into organic molecules, targeting specifically amino acids and peptides. We followed the CNO^-^ (m/z 42) and C_3_N^-^ (m/z 50) peaks which, along with CN^-^, were the three most intense peaks in the samples and are characteristic of protein fragmentation (Chen et al., 2011), commonly used in negative ToF-SIMS analyses (Sanni et al., 2002; Wagner et al., 2002; Sjövall et al., 2006). However, deconvolution interferences, caused by neighboring hydrocarbon peaks, induced an overestimation of ^15^N enrichment into peptides by approximately 4 Atom% (Figure 3). This issue was specific to the CNO^-^ and C_3_N^-^ peaks and did not impact previous quantification of ^12^C^15^N and ^12^C^14^N peaks (Figure 1). Although the signals exhibited an overall increase through time, these deconvolution artifacts prevented an accurate quantification of the proportion of ^15^N channeled by *Synechococcus* toward amino-acid synthesis. Besides CNO^-^ and C_3_N^-^, we were not able to detect or reliably quantify other ^15^N-labeled organic nitrogen compounds in *Synechococcus* cells. Prior studies of biological material using ToF-SIMS used positive polarity, which enables a degree of separation for specific amino acids in the mass spectrum. Since we were focusing on comparing the two SIMS techniques, we used the negative polarity mass spectra to image and quantify CN^-^ ions. However, our data clearly show that using negative polarity provides insufficient information to identify specific amino acids.

## Conclusion

The three instruments do not require extensive sample preparation to analyze stable isotope incorporation in microbial populations. EA-IRMS provides quick, accurate and relatively inexpensive quantification but is restricted to large sample amounts: the dried biomass needed for analysis should be higher than 0.15 mg per sample, which is equivalent to the microbial biomass present in hundreds of milliliters of oceanic water. Conversely, SIMS methods require longer analysis, but can push measurements beyond the population average, unraveling cellular heterogeneity and enabling access to complex biological processes, or rare microorganisms (Musat et al., 2008; Zimmermann et al., 2015). Currently, NanoSIMS is the only instrument that allows the visualization and quantification of stable isotope tracers within any cell type (from multicellular organisms to virus particles). In comparison, the use of ToF-SIMS has been limited in microbiology, mainly because of its lower spatial resolution, however, our data clearly show that this instrument enables the detection of isotopic enrichment in cells as small as 2 μm. Although peak overlap and asymmetry can prevent accurate isotopic quantification when enrichment levels are very low, this instrument can reliably detect microscale enrichments of ^12^C^15^N as soon as they exceed 0.2 Atom% (Figure 1 and Supplementary Table 2). Therefore, ToF-SIMS is a useful technique for detecting enrichment in biological samples, especially if researchers do not have access to a NanoSIMS. Recent technological developments – increasing ToF-SIMS resolution to less than 200 nm for inorganic molecules and less than 2 μm for organic compounds (Passarelli et al., 2017) – will undoubtedly increase the relevance of this instrument to study microbial interactions. SIMS approaches hold great potential to unravel some more intricate aspects of *Synechococcus* ecology by quantifying more accurately fluxes of the major elements, localizing where these elements are stored intracellularly and in which form, and visualizing how these cells interact with other microbes such as heterotrophic bacteria or zooplankton. Future research coupling the approaches used here to examine the dynamics of single cell ecophysiology will deliver more precise insights into the metabolic interactions of microbes at the micro-scale, which in-turn promises to contribute to a clearer understanding of the importance of microbial processes in ocean-scale chemical fluxes.

## Author Contributions

MG, JS, MP, and J-BR conceived and designed the study. MG, PG, and PC carried out the NanoSIMS data acquisition. SC and MG carried out the ToF-SIMS data acquisition. CM carried out the peak deconvolution. MG and RP carried out the EA-IRMS data acquisition. MG and J-BR drafted the manuscript. All authors read and approved the final manuscript.

## Conflict of Interest Statement

The authors declare that the research was conducted in the absence of any commercial or financial relationships that could be construed as a potential conflict of interest.

## Footnotes

1 http://fiji.sc/Fiji

2 http://nrims.harvard.edu/software
