# Supplementary material for: Quantifying Inorganic Nitrogen Assimilation by Synechococcus Using Bulk and Single-Cell Mass Spectrometry: A Comparative Study
Source: Front Microbiol. 2018 Nov 27;9:2847. doi: 10.3389/fmicb.2018.02847 (PMC6277480; doi:10.3389/fmicb.2018.02847)
Supplement: Supplementary file 1 [file Table_1.docx]

**Supplementary Material**

**Quantifying inorganic nitrogen assimilation by *Synechococcus* using bulk and single-cell mass spectrometry: a comparative study**

Marco Giardina, Soshan Cheong, Christopher E. Marjo, Peta L. Clode, Paul Guagliardo, Russell Pickford, Mathieu Pernice^*^, Justin R. Seymour, Jean-Baptiste Raina^*^

^*^**Correspondence:**

Mathieu Pernice

[Mathieu.Pernice@uts.edu.au](mailto:Mathieu.Pernice@uts.edu.au)

Jean-Baptiste Raina

[Jean-Baptiste.Raina@uts.edu.au](mailto:Justin.Seymour@uts.edu.au)

**Supplementary Figure 1.** Dilution series of glutamic acid standards (with increasing proportion of ^15^N) measured with EA-IRMS.

**Supplementary Figure 2.** Examples of ToF-SIMS spectra showing quality of asymmetric peak fitting for sample T1: (a) typical high-quality fit (standard error 0.993) observed when peak heights have sufficient counts (>20) and, (b) poor quality fit (standard error 0.884) where peak height <20 counts and approaching detection limit of the instrument. Peak masses are shown, along with the accumulated counts beneath each peak. Note ^11^B^16^O^-^ at m/z 27.00422 cannot be resolved.

**Supplementary Table 1.** Pairwise comparison of negative control (T_0_) against 15 minutes (T_1_) of each respective instrument with T-Test (EA-IRMS) and Mann-Whitney U-test (NanoSIMS and ToF-SIMS)

| Method | Pairwise comparison | Test | U-value | *t* | *p*-value |
| --- | --- | --- | --- | --- | --- |
| EA-IRMS | Neg. Contr./15 min | t-test | - | -51.42 | 0.000 |
| ToF-SIMS | Neg. Contr./15 min | Mann-Whitney | 133 | - | 0.002 |
| NanoSIMS | Neg. Contr./15 min | Mann-Whitney | 44 | - | 0.000 |

**Supplementary Table 2.** Summary of Kruskal-Wallis test and Dunn’s post hoc test with Bonferroni adjustment. Replicate for SIMS correspond to single cells. *Only two replicates were analyzed for T_5_ with EA-IRMS as one replicate was lost.

| Sample | Replicates | Mean  (At%) | SE | Kruskal-Wallis test  *p*-value | Post hoc pairwise comparisons | Test Statistic | Std. Error | Std. Test Statistic | Bonferroni Adjusted *p*-value |
| --- | --- | --- | --- | --- | --- | --- | --- | --- | --- |
| T_0_ | EA-IRMS = 3  ToF-SIMS = 23  NanoSIMS = 50 | 0.371  0.460  0.374 | 0.001  0.016  0.001 | 0.000 | EA-IRMS/ToF-SIMS  ToF-SIMS/NanoSIMS  EA-IRMS/NanoSIMS | 28.198  -36.145  -7.947 | 13.556  5.564  13.127 | -2.666  5.068  -0.605 | 0.023  0.000  1 |
| 15 min | EA-IRMS = 3  ToF-SIMS = 25  NanoSIMS = 56 | 0.516  0.543  0.481 | 0.002  0.017  0.012 | 0.006 | EA-IRMS/ToF-SIMS  ToF-SIMS/NanoSIMS  EA-IRMS/NanoSIMS | -3.833  18.321  14.488 | 14.761  5.881  14.284 | -0.260  3.115  1.014 | 1  0.006  0.931 |
| 30 min | EA-IRMS = 3  ToF-SIMS = 29  NanoSIMS = 58 | 0.700  0.751  0.642 | 0.005  0.024  0.024 | 0.010 | EA-IRMS/ToF-SIMS  ToF-SIMS/NanoSIMS  EA-IRMS/NanoSIMS | -7.310  17.948  10.638 | 15.844  5.942  15.468 | -0.461  3.021  0.688 | 1  0.008  1 |
| 60 minutes | EA-IRMS = 3  ToF-SIMS = 18  NanoSIMS = 59 | 1.273  1.242  0.920 | 0.006  0.043  0.050 | 0.000 | EA-IRMS/ToF-SIMS  ToF-SIMS/NanoSIMS  EA-IRMS/NanoSIMS | 2.278  24.824  27.102 | 14.491  6.257  13.753 | 0.157  3.967  1.971 | 1  0.000  0.146 |
| 120 minutes | EA-IRMS = 3  ToF-SIMS = 19  NanoSIMS = 59 | 2.995  2.869  1.965 | 0.009  0.176  0.099 | 0.000 | EA-IRMS/ToF-SIMS  ToF-SIMS/NanoSIMS  EA-IRMS/NanoSIMS | 7.368  23.208  30.576 | 14.616  6.206  13.924 | 0.504  3.740  2.196 | 1  0.001  0.084 |
| 240 minutes | EA-IRMS = 2*  ToF-SIMS = 20  NanoSIMS = 53 | 7.460  5.898  5.225 | 0.006  0.383  0.266 | 0.051 | EA-IRMS/ToF-SIMS  ToF-SIMS/NanoSIMS  EA-IRMS/NanoSIMS | NA  NA  NA | NA  NA  NA | NA  NA  NA | NA  NA  NA |
| 360 minutes | EA-IRMS = 3  ToF-SIMS = 52  NanoSIMS = 71 | 12.887  11.608  9.219 | 0.057  0.587  0.375 | 0.000 | EA-IRMS/ToF-SIMS  ToF-SIMS/NanoSIMS  EA-IRMS/NanoSIMS | 26.513  23.576  50.089 | 21.683  6.665  21.524 | 1.223  3.537  2.327 | 0.664  0.001  0.060 |

**Supplementary Table 3.** Raw NanoSIMS data.

| **Time** | **File name** | **ROI name** | **^12^C^14^N sum** | **^12^C^15^N sum** | **Ratio** | **AT%** |
| --- | --- | --- | --- | --- | --- | --- |
| T_0_ | 030517_neg_1.nrrd | Synech_1 | 443740 | 1681 | 0.003788254 | 0.377395767 |
| T_0_ | 030517_neg_1.nrrd | Synech_2 | 803093 | 3044 | 0.003790346 | 0.377603311 |
| T_0_ | 030517_neg_2.nrrd | Synech_1 | 871776 | 3264 | 0.003744081 | 0.373011519 |
| T_0_ | 030517_neg_2.nrrd | Synech_2 | 806267 | 3041 | 0.003771703 | 0.375753113 |
| T_0_ | 030517_neg_2.nrrd | Synech_3 | 423367 | 1642 | 0.003878432 | 0.38634476 |
| T_0_ | 030517_neg_2.nrrd | Synech_4 | 411521 | 1559 | 0.003788385 | 0.377408734 |
| T_0_ | 030517_neg_2.nrrd | Synech_5 | 433481 | 1591 | 0.003670288 | 0.365686599 |
| T_0_ | 030517_neg_2.nrrd | Synech_6 | 785447 | 2996 | 0.003814388 | 0.379989422 |
| T_0_ | 030517_neg_2.nrrd | Synech_7 | 405248 | 1448 | 0.003573121 | 0.356039892 |
| T_0_ | 030517_neg_2.nrrd | Synech_8 | 363295 | 1386 | 0.003815081 | 0.380058188 |
| T_0_ | 030517_neg_2.nrrd | Synech_9 | 361647 | 1342 | 0.003710801 | 0.369708173 |
| T_0_ | 030517_neg_3.nrrd | Synech_1 | 676901 | 2605 | 0.003848421 | 0.38336674 |
| T_0_ | 030517_neg_3.nrrd | Synech_2 | 498122 | 1921 | 0.003856485 | 0.384166962 |
| T_0_ | 030517_neg_3.nrrd | Synech_3 | 678514 | 2545 | 0.003750844 | 0.37368275 |
| T_0_ | 030517_neg_3.nrrd | Synech_4 | 516249 | 1938 | 0.003754002 | 0.37399626 |
| T_0_ | 030517_neg_3.nrrd | Synech_5 | 792947 | 2994 | 0.003775788 | 0.376158534 |
| T_0_ | 030517_neg_3.nrrd | Synech_6 | 763535 | 2918 | 0.003821698 | 0.380714799 |
| T_0_ | 030517_neg_4.nrrd | Synech_1 | 554061 | 2131 | 0.003846147 | 0.383141074 |
| T_0_ | 030517_neg_4.nrrd | Synech_2 | 522771 | 1943 | 0.003716733 | 0.370296962 |
| T_0_ | 030517_neg_4.nrrd | Synech_3 | 497076 | 1919 | 0.003860577 | 0.384572992 |
| T_0_ | 030517_neg_4.nrrd | Synech_4 | 393206 | 1503 | 0.003822424 | 0.380786858 |
| T_0_ | 030517_neg_4.nrrd | Synech_5 | 505725 | 1879 | 0.003715458 | 0.370170448 |
| T_0_ | 030517_neg_4.nrrd | Synech_6 | 466190 | 1734 | 0.003719514 | 0.370572999 |
| T_0_ | 030517_neg_4.nrrd | Synech_7 | 553694 | 2051 | 0.003704212 | 0.369054153 |
| T_0_ | 030517_neg_5.nrrd | Synech_1 | 855039 | 3167 | 0.003703925 | 0.369025619 |
| T_0_ | 030517_neg_5.nrrd | Synech_2 | 685042 | 2616 | 0.003818744 | 0.380421663 |
| T_0_ | 030517_neg_5.nrrd | Synech_3 | 999455 | 3662 | 0.003663997 | 0.365062101 |
| T_0_ | 030517_neg_5.nrrd | Synech_4 | 911569 | 3559 | 0.003904257 | 0.388907344 |
| T_0_ | 030517_neg_5.nrrd | Synech_5 | 539484 | 2010 | 0.003725782 | 0.371195249 |
| T_0_ | 030517_neg_5.nrrd | Synech_6 | 948301 | 3543 | 0.003736156 | 0.37222486 |
| T_0_ | 030517_neg_6.nrrd | Synech_1 | 540284 | 2055 | 0.003803555 | 0.378914295 |
| T_0_ | 030517_neg_6.nrrd | Synech_10 | 535694 | 1955 | 0.003649472 | 0.363620131 |
| T_0_ | 030517_neg_6.nrrd | Synech_11 | 193188 | 707 | 0.003659648 | 0.364630341 |
| T_0_ | 030517_neg_6.nrrd | Synech_2 | 281063 | 1055 | 0.003753607 | 0.373956997 |
| T_0_ | 030517_neg_6.nrrd | Synech_3 | 463707 | 1698 | 0.003661795 | 0.364843523 |
| T_0_ | 030517_neg_6.nrrd | Synech_4 | 485036 | 1873 | 0.003861569 | 0.384671468 |
| T_0_ | 030517_neg_6.nrrd | Synech_5 | 295359 | 1127 | 0.003815695 | 0.380119129 |
| T_0_ | 030517_neg_6.nrrd | Synech_6 | 461839 | 1690 | 0.003659284 | 0.364594233 |
| T_0_ | 030517_neg_6.nrrd | Synech_7 | 404315 | 1506 | 0.003724819 | 0.371099573 |
| T_0_ | 030517_neg_6.nrrd | Synech_8 | 444881 | 1662 | 0.00373583 | 0.3721926 |
| T_0_ | 030517_neg_6.nrrd | Synech_9 | 475237 | 1756 | 0.003694998 | 0.368139574 |
| T_0_ | 030517_neg_7.nrrd | Synech_1 | 1478083 | 5573 | 0.003770424 | 0.375626156 |
| T_0_ | 030517_neg_7.nrrd | Synech_2 | 1772625 | 6583 | 0.003713701 | 0.369996088 |
| T_0_ | 030517_neg_7.nrrd | Synech_3 | 1414467 | 5347 | 0.003780223 | 0.376598625 |
| T_0_ | 030517_neg_7.nrrd | Synech_4 | 1641877 | 6090 | 0.003709169 | 0.369546235 |
| T_0_ | 030517_neg_8.nrrd | Synech_1 | 377023 | 1435 | 0.003806134 | 0.379170212 |
| T_0_ | 030517_neg_8.nrrd | Synech_2 | 615952 | 2248 | 0.003649635 | 0.363636364 |
| T_0_ | 030517_neg_8.nrrd | Synech_3 | 626084 | 2410 | 0.003849324 | 0.383456326 |
| T_0_ | 030517_neg_8.nrrd | Synech_4 | 591072 | 2229 | 0.003771114 | 0.375694631 |
| T_0_ | 030517_neg_8.nrrd | Synech_5 | 512217 | 1877 | 0.003664463 | 0.365108326 |
| 15 minutes | 020517_T1_chain_1.nrrd | Synech_1 | 691703 | 4723 | 0.006828075 | 0.678176863 |
| 15 minutes | 020517_T1_chain_1.nrrd | Synech_2 | 834195 | 5709 | 0.006843724 | 0.679720539 |
| 15 minutes | 020517_T1_chain_1.nrrd | Synech_3 | 787861 | 3999 | 0.005075768 | 0.505013512 |
| 15 minutes | 020517_T1_chain_1.nrrd | Synech_4 | 555381 | 2591 | 0.004665266 | 0.464360219 |
| 15 minutes | 020517_T1_chain_1.nrrd | Synech_5 | 917883 | 3761 | 0.004097472 | 0.408075135 |
| 15 minutes | 020517_T1_chain_1.nrrd | Synech_6 | 677739 | 2931 | 0.004324674 | 0.430605139 |
| 15 minutes | 020517_T1_chain_1.nrrd | Synech_7 | 496461 | 2087 | 0.004203754 | 0.41861566 |
| 15 minutes | 020517_T1_chain_1.nrrd | Synech_8 | 715672 | 2904 | 0.004057725 | 0.404132618 |
| 15 minutes | 020517_T1_chain_2.nrrd | Synech_1 | 556824 | 2284 | 0.004101835 | 0.408507837 |
| 15 minutes | 020517_T1_chain_2.nrrd | Synech_2 | 668449 | 3012 | 0.004505953 | 0.44857408 |
| 15 minutes | 020517_T1_chain_2.nrrd | Synech_3 | 618051 | 2798 | 0.004527134 | 0.450673191 |
| 15 minutes | 020517_T1_chain_3.nrrd | Synech_1 | 564094 | 2196 | 0.003892968 | 0.387787176 |
| 15 minutes | 020517_T1_chain_3.nrrd | Synech_2 | 785578 | 4173 | 0.005312012 | 0.528394393 |
| 15 minutes | 020517_T1_chain_3.nrrd | Synech_3 | 717831 | 2850 | 0.003970294 | 0.395459295 |
| 15 minutes | 020517_T1_chain_4.nrrd | Synech_1 | 539904 | 2904 | 0.005378734 | 0.5349958 |
| 15 minutes | 020517_T1_chain_4.nrrd | Synech_2 | 952044 | 4356 | 0.004575419 | 0.455457967 |
| 15 minutes | 020517_T1_chain_5.nrrd | Synech_1 | 531011 | 2409 | 0.004536629 | 0.451614113 |
| 15 minutes | 020517_T1_chain_5.nrrd | Synech_2 | 773961 | 2958 | 0.003821898 | 0.380734671 |
| 15 minutes | 020517_T1_chain_6.nrrd | Synech_1 | 762262 | 4337 | 0.005689645 | 0.565745585 |
| 15 minutes | 020517_T1_chain_6.nrrd | Synech_2 | 770877 | 5517 | 0.007156784 | 0.710592818 |
| 15 minutes | 020517_T1_chain_6.nrrd | Synech_3 | 677839 | 2779 | 0.004099794 | 0.408305393 |
| 15 minutes | 020517_T1_chain_7.nrrd | Synech_1 | 560919 | 2413 | 0.004301869 | 0.428344209 |
| 15 minutes | 020517_T1_chain_7.nrrd | Synech_2 | 534388 | 2414 | 0.004517317 | 0.449700262 |
| 15 minutes | 020517_T1_chain_7.nrrd | Synech_3 | 1074291 | 7871 | 0.007326693 | 0.727340269 |
| 15 minutes | 020517_T1_chain_8.nrrd | Synech_1 | 889214 | 3738 | 0.004203712 | 0.418611527 |
| 15 minutes | 020517_T1_chain_8.nrrd | Synech_2 | 777940 | 5174 | 0.006650899 | 0.660695633 |
| 15 minutes | 020517_T1_chain_8.nrrd | Synech_3 | 553651 | 2294 | 0.004143404 | 0.412630746 |
| 15 minutes | 020517_T1_chain_9.nrrd | Synech_1 | 885380 | 3461 | 0.003909056 | 0.389383478 |
| 15 minutes | 020517_T1_chain_9.nrrd | Synech_2 | 453531 | 2046 | 0.004511268 | 0.449100811 |
| 15 minutes | 020517_T1_chain_10.nrrd | Synech_1 | 827493 | 3838 | 0.004638106 | 0.461669299 |
| 15 minutes | 020517_T1_chain_10.nrrd | Synech_2 | 477678 | 1942 | 0.0040655 | 0.404903882 |
| 15 minutes | 020517_T1_chain_10.nrrd | Synech_3 | 528944 | 2114 | 0.003996642 | 0.39807328 |
| 15 minutes | 020517_T1_chain_11.nrrd | Synech_1 | 374297 | 1856 | 0.004958629 | 0.493416243 |
| 15 minutes | 020517_T1_chain_11.nrrd | Synech_2 | 588454 | 3514 | 0.00597158 | 0.593613168 |
| 15 minutes | 020517_T1_chain_12.nrrd | Synech_1 | 727501 | 3004 | 0.004129204 | 0.411222374 |
| 15 minutes | 020517_T1_chain_12.nrrd | Synech_2 | 1005091 | 6239 | 0.006207398 | 0.616910405 |
| 15 minutes | 020517_T1_chain_12.nrrd | Synech_3 | 836621 | 4300 | 0.005139723 | 0.51134411 |
| 15 minutes | 050517_T1_chain_1.nrrd | Synech_1 | 729187 | 4645 | 0.006370108 | 0.632978665 |
| 15 minutes | 050517_T1_chain_1.nrrd | Synech_2 | 819560 | 4466 | 0.005449265 | 0.54197319 |
| 15 minutes | 050517_T1_chain_1.nrrd | Synech_3 | 698868 | 3758 | 0.005377267 | 0.534850689 |
| 15 minutes | 050517_T1_chain_1.nrrd | Synech_4 | 563600 | 2712 | 0.004811923 | 0.478887963 |
| 15 minutes | 050517_T1_chain_1.nrrd | Synech_5 | 1293540 | 5548 | 0.004289005 | 0.427068836 |
| 15 minutes | 050517_T1_chain_1.nrrd | Synech_6 | 562442 | 2890 | 0.005138308 | 0.511204036 |
| 15 minutes | 050517_T1_chain_2.nrrd | Synech_1 | 1146761 | 4989 | 0.004350514 | 0.43316692 |
| 15 minutes | 050517_T1_chain_2.nrrd | Synech_2 | 699166 | 2626 | 0.003755903 | 0.374184944 |
| 15 minutes | 050517_T1_chain_3.nrrd | Synech_1 | 1099588 | 4250 | 0.003865084 | 0.385020266 |
| 15 minutes | 050517_T1_chain_3.nrrd | Synech_2 | 870793 | 4900 | 0.005627055 | 0.559556831 |
| 15 minutes | 050517_T1_chain_4.nrrd | Synech_1 | 1143819 | 5355 | 0.004681685 | 0.465986874 |
| 15 minutes | 050517_T1_chain_4.nrrd | Synech_2 | 858830 | 3358 | 0.003909971 | 0.389474221 |
| 15 minutes | 050517_T1_chain_4.nrrd | Synech_3 | 953473 | 4240 | 0.004446901 | 0.442721358 |
| 15 minutes | 050517_T1_chain_5.nrrd | Synech_1 | 831237 | 3692 | 0.004441573 | 0.442193288 |
| 15 minutes | 050517_T1_chain_5.nrrd | Synech_2 | 930679 | 5253 | 0.005644266 | 0.561258724 |
| 15 minutes | 050517_T1_chain_5.nrrd | Synech_3 | 931636 | 4056 | 0.004353632 | 0.433475973 |
| 15 minutes | 050517_T1_chain_5.nrrd | Synech_4 | 710617 | 3892 | 0.005476931 | 0.544709724 |
| 15 minutes | 050517_T1_chain_6.nrrd | Synech_1 | 969736 | 3722 | 0.003838158 | 0.382348288 |
| 15 minutes | 050517_T1_chain_6.nrrd | Synech_2 | 768388 | 3437 | 0.004473001 | 0.445308198 |
| 30 minutes | 050517_T2_chain_1.nrrd | Synech_1 | 917437 | 4579 | 0.004991078 | 0.496629126 |
| 30 minutes | 050517_T2_chain_1.nrrd | Synech_2 | 790274 | 4282 | 0.005418374 | 0.538917332 |
| 30 minutes | 050517_T2_chain_1.nrrd | Synech_3 | 812123 | 4009 | 0.004936444 | 0.491219558 |
| 30 minutes | 050517_T2_chain_1.nrrd | Synech_4 | 958716 | 9524 | 0.00993412 | 0.98364042 |
| 30 minutes | 050517_T2_chain_2.nrrd | Synech_1 | 623019 | 5287 | 0.008486098 | 0.841468966 |
| 30 minutes | 050517_T2_chain_2.nrrd | Synech_2 | 867700 | 5328 | 0.006140371 | 0.610289704 |
| 30 minutes | 050517_T2_chain_3.nrrd | Synech_1 | 823528 | 4151 | 0.005040509 | 0.501522933 |
| 30 minutes | 050517_T2_chain_3.nrrd | Synech_2 | 791392 | 4561 | 0.005763263 | 0.573023784 |
| 30 minutes | 050517_T2_chain_4.nrrd | Synech_1 | 1032165 | 4822 | 0.004671734 | 0.465001008 |
| 30 minutes | 050517_T2_chain_4.nrrd | Synech_2 | 672522 | 2930 | 0.004356735 | 0.4337836 |
| 30 minutes | 050517_T2_chain_5.nrrd | Synech_1 | 855900 | 6381 | 0.00745531 | 0.740013986 |
| 30 minutes | 050517_T2_chain_5.nrrd | Synech_2 | 810689 | 4730 | 0.005834543 | 0.580069878 |
| 30 minutes | 050517_T2_chain_6.nrrd | Synech_1 | 786014 | 4930 | 0.006272153 | 0.623305822 |
| 30 minutes | 050517_T2_chain_6.nrrd | Synech_2 | 878528 | 8792 | 0.010007649 | 0.990848848 |
| 30 minutes | 050517_T2_chain_6.nrrd | Synech_3 | 1438264 | 13277 | 0.009231268 | 0.914683085 |
| 30 minutes | 050517_T2_chain_7.nrrd | Synech_1 | 1016265 | 4646 | 0.004571642 | 0.455083744 |
| 30 minutes | 050517_T2_chain_7.nrrd | Synech_2 | 644635 | 5439 | 0.008437333 | 0.836673979 |
| 30 minutes | 050517_T2_chain_7.nrrd | Synech_3 | 590597 | 3372 | 0.005709477 | 0.567706395 |
| 30 minutes | 050517_T2_chain_8.nrrd | Synech_1 | 1040671 | 5834 | 0.005605998 | 0.557474642 |
| 30 minutes | 050517_T2_chain_8.nrrd | Synech_2 | 1180827 | 7290 | 0.006173639 | 0.613575936 |
| 30 minutes | 050517_T2_chain_9.nrrd | Synech_1 | 714628 | 3264 | 0.004567411 | 0.45466449 |
| 30 minutes | 050517_T2_chain_9.nrrd | Synech_2 | 884776 | 3727 | 0.004212366 | 0.419469602 |
| 30 minutes | 050517_T2_chain_9.nrrd | Synech_3 | 748021 | 4997 | 0.006680294 | 0.663596355 |
| 30 minutes | 050517_T2_chain_9.nrrd | Synech_4 | 827144 | 5225 | 0.006316917 | 0.627726405 |
| 30 minutes | 050517_T2_chain_9.nrrd | Synech_5 | 846400 | 4062 | 0.004799149 | 0.477622751 |
| 30 minutes | 050517_T2_chain_9.nrrd | Synech_6 | 957788 | 8985 | 0.00938099 | 0.929380527 |
| 30 minutes | 050517_T2_chain_9.nrrd | Synech_7 | 767450 | 6061 | 0.007897583 | 0.783569982 |
| 30 minutes | 050517_T2_chain_10.nrrd | Synech_1 | 730817 | 3657 | 0.005003989 | 0.497907346 |
| 30 minutes | 050517_T2_chain_11.nrrd | Synech_1 | 1055033 | 7271 | 0.006891728 | 0.684455674 |
| 30 minutes | 050517_T2_chain_11.nrrd | Synech_2 | 791467 | 6976 | 0.008814012 | 0.873700439 |
| 30 minutes | 050517_T2_chain_11.nrrd | Synech_3 | 1015595 | 5282 | 0.005200892 | 0.517398276 |
| 30 minutes | 050517_T2_chain_12.nrrd | Synech_1 | 1571988 | 6883 | 0.004378532 | 0.435944419 |
| 30 minutes | 050517_T2_chain_12.nrrd | Synech_2 | 822180 | 7366 | 0.008959109 | 0.887955581 |
| 30 minutes | 050517_T2_chain_12.nrrd | Synech_3 | 732271 | 6387 | 0.008722181 | 0.86467621 |
| 30 minutes | 050517_T2_chain_13.nrrd | Synech_1 | 1085764 | 7946 | 0.007318349 | 0.726517998 |
| 30 minutes | 050517_T2_chain_13.nrrd | Synech_2 | 772178 | 3392 | 0.00439277 | 0.437355751 |
| 30 minutes | 050517_T2_chain_13.nrrd | Synech_3 | 1430749 | 6358 | 0.004443826 | 0.442416605 |
| 30 minutes | 050517_T2_chain_14.nrrd | Synech_1 | 1244105 | 8432 | 0.006777563 | 0.673193686 |
| 30 minutes | 050517_T2_chain_14.nrrd | Synech_2 | 950515 | 4519 | 0.004754265 | 0.473176871 |
| 30 minutes | 050517_T2_chain_15.nrrd | Synech_1 | 532775 | 2702 | 0.005071559 | 0.504596836 |
| 30 minutes | 050517_T2_chain_15.nrrd | Synech_2 | 835353 | 4653 | 0.0055701 | 0.553924615 |
| 30 minutes | 050517_T2_chain_16.nrrd | Synech_1 | 1007888 | 4508 | 0.004472719 | 0.445280305 |
| 30 minutes | 050517_T2_chain_16.nrrd | Synech_2 | 961443 | 5417 | 0.005634239 | 0.560267257 |
| 30 minutes | 050517_T2_chain_16.nrrd | Synech_3 | 785979 | 3534 | 0.004496303 | 0.447617709 |
| 30 minutes | 050517_T2_chain_17.nrrd | Synech_1 | 1229300 | 11335 | 0.009220695 | 0.913645029 |
| 30 minutes | 050517_T2_chain_17.nrrd | Synech_2 | 532287 | 3833 | 0.007201002 | 0.714951876 |
| 30 minutes | 050517_T2_chain_17.nrrd | Synech_3 | 322694 | 4109 | 0.012733425 | 1.257332399 |
| 30 minutes | 050517_T2_chain_17.nrrd | Synech_4 | 427296 | 3053 | 0.00714493 | 0.709424212 |
| 30 minutes | 050517_T2_chain_18.nrrd | Synech_1 | 914885 | 5408 | 0.005911125 | 0.587638937 |
| 30 minutes | 050517_T2_chain_18.nrrd | Synech_2 | 748075 | 4130 | 0.005520837 | 0.549052452 |
| 30 minutes | 050517_T2_chain_18.nrrd | Synech_3 | 543671 | 3040 | 0.005591617 | 0.556052466 |
| 30 minutes | 050517_T2_chain_19.nrrd | Synech_1 | 572185 | 3841 | 0.006712864 | 0.666810179 |
| 30 minutes | 050517_T2_chain_19.nrrd | Synech_2 | 915498 | 8184 | 0.008939397 | 0.886019214 |
| 30 minutes | 050517_T2_chain_20.nrrd | Synech_1 | 893040 | 6793 | 0.007606602 | 0.754917857 |
| 30 minutes | 050517_T2_chain_20.nrrd | Synech_2 | 702388 | 5307 | 0.007555653 | 0.749899321 |
| 30 minutes | 050517_T2_chain_20.nrrd | Synech_3 | 933397 | 5341 | 0.00572211 | 0.568955342 |
| 30 minutes | 050517_T2_chain_20.nrrd | Synech_4 | 609063 | 4690 | 0.007700353 | 0.764151051 |
| 30 minutes | 050517_T2_chain_20.nrrd | Synech_5 | 699915 | 2747 | 0.003924762 | 0.390941875 |
| 60 minutes | 010517_T3_chain_1.nrrd | Synech_1 | 647924 | 6690 | 0.010325285 | 1.02197631 |
| 60 minutes | 010517_T3_chain_1.nrrd | Synech_2 | 689915 | 9530 | 0.013813296 | 1.362508846 |
| 60 minutes | 010517_T3_chain_2.nrrd | Synech_1 | 572716 | 2219 | 0.003874521 | 0.385956673 |
| 60 minutes | 010517_T3_chain_2.nrrd | Synech_2 | 578893 | 7015 | 0.012117956 | 1.197286946 |
| 60 minutes | 010517_T3_chain_3.nrrd | Synech_1 | 677687 | 6311 | 0.009312559 | 0.922663517 |
| 60 minutes | 010517_T3_chain_3.nrrd | Synech_2 | 713957 | 11676 | 0.016353926 | 1.609077867 |
| 60 minutes | 010517_T3_chain_3.nrrd | Synech_3 | 635943 | 4855 | 0.007634332 | 0.757649056 |
| 60 minutes | 010517_T3_chain_4.nrrd | Synech_1 | 493373 | 4342 | 0.008800644 | 0.872386808 |
| 60 minutes | 010517_T3_chain_4.nrrd | Synech_2 | 665701 | 10426 | 0.015661686 | 1.542017994 |
| 60 minutes | 010517_T3_chain_5.nrrd | Synech_1 | 772056 | 8981 | 0.011632576 | 1.149881504 |
| 60 minutes | 010517_T3_chain_5.nrrd | Synech_2 | 370036 | 9263 | 0.0250327 | 2.442136678 |
| 60 minutes | 010517_T3_chain_5.nrrd | Synech_3 | 808225 | 6301 | 0.007796096 | 0.773578744 |
| 60 minutes | 010517_T3_chain_5.nrrd | Synech_4 | 453817 | 4865 | 0.01072018 | 1.060647682 |
| 60 minutes | 010517_T3_chain_5.nrrd | Synech_5 | 533476 | 9147 | 0.017146038 | 1.685700754 |
| 60 minutes | 010517_T3_chain_6.nrrd | Synech_1 | 592647 | 4784 | 0.008072259 | 0.800761929 |
| 60 minutes | 010517_T3_chain_6.nrrd | Synech_2 | 712567 | 7531 | 0.010568831 | 1.045829873 |
| 60 minutes | 010517_T3_chain_6.nrrd | Synech_3 | 659773 | 10701 | 0.016219215 | 1.596035044 |
| 60 minutes | 010517_T3_chain_7.nrrd | Synech_1 | 600738 | 6296 | 0.010480442 | 1.037174195 |
| 60 minutes | 010517_T3_chain_7.nrrd | Synech_2 | 606076 | 3149 | 0.005195718 | 0.516886208 |
| 60 minutes | 010517_T3_chain_7.nrrd | Synech_3 | 641757 | 4462 | 0.006952787 | 0.690477996 |
| 60 minutes | 010517_T3_chain_8.nrrd | Synech_1 | 668813 | 5488 | 0.008205582 | 0.813879855 |
| 60 minutes | 010517_T3_chain_8.nrrd | Synech_10 | 699899 | 3386 | 0.004837841 | 0.481454887 |
| 60 minutes | 010517_T3_chain_8.nrrd | Synech_2 | 524012 | 5090 | 0.009713518 | 0.962007326 |
| 60 minutes | 010517_T3_chain_8.nrrd | Synech_3 | 829071 | 3319 | 0.004003276 | 0.398731364 |
| 60 minutes | 010517_T3_chain_8.nrrd | Synech_4 | 668130 | 5709 | 0.008544744 | 0.847235022 |
| 60 minutes | 010517_T3_chain_8.nrrd | Synech_5 | 601951 | 7115 | 0.011819899 | 1.168182102 |
| 60 minutes | 010517_T3_chain_8.nrrd | Synech_6 | 692036 | 9617 | 0.013896676 | 1.37062052 |
| 60 minutes | 010517_T3_chain_8.nrrd | Synech_7 | 415003 | 3182 | 0.007667414 | 0.760907254 |
| 60 minutes | 010517_T3_chain_8.nrrd | Synech_8 | 445559 | 5486 | 0.012312623 | 1.216286623 |
| 60 minutes | 010517_T3_chain_8.nrrd | Synech_9 | 337676 | 2344 | 0.006941565 | 0.689371213 |
| 60 minutes | 010517_T3_chain_9.nrrd | Synech_1 | 522154 | 7537 | 0.014434439 | 1.422905052 |
| 60 minutes | 010517_T3_chain_9.nrrd | Synech_2 | 473870 | 2877 | 0.006071285 | 0.603464731 |
| 60 minutes | 010517_T3_chain_9.nrrd | Synech_3 | 600588 | 4189 | 0.006974831 | 0.692652002 |
| 60 minutes | 010517_T3_chain_10.nrrd | Synech_1 | 524996 | 6419 | 0.01222676 | 1.207907191 |
| 60 minutes | 010517_T3_chain_10.nrrd | Synech_2 | 733565 | 6322 | 0.008618187 | 0.854454802 |
| 60 minutes | 010517_T3_chain_10.nrrd | Synech_3 | 801492 | 3711 | 0.004630115 | 0.460877568 |
| 60 minutes | 010517_T3_chain_10.nrrd | Synech_4 | 466786 | 4416 | 0.009460438 | 0.937177686 |
| 60 minutes | 010517_T3_chain_11.nrrd | Synech_1 | 631135 | 6860 | 0.010869307 | 1.075243536 |
| 60 minutes | 010517_T3_chain_11.nrrd | Synech_2 | 733118 | 6763 | 0.009224982 | 0.914065911 |
| 60 minutes | 010517_T3_chain_11.nrrd | Synech_3 | 679385 | 4432 | 0.006523547 | 0.648126619 |
| 60 minutes | 010517_T3_chain_12.nrrd | Synech_1 | 456968 | 5321 | 0.011644141 | 1.151011597 |
| 60 minutes | 010517_T3_chain_12.nrrd | Synech_2 | 613281 | 5036 | 0.00821157 | 0.814468954 |
| 60 minutes | 010517_T3_chain_12.nrrd | Synech_3 | 522412 | 4383 | 0.00838993 | 0.832012453 |
| 60 minutes | 010517_T3_chain_14.nrrd | Synech_1 | 357517 | 3340 | 0.009342213 | 0.925574397 |
| 60 minutes | 010517_T3_chain_14.nrrd | Synech_2 | 719710 | 2160 | 0.003001209 | 0.299222852 |
| 60 minutes | 010517_T3_chain_15.nrrd | Synech_1 | 880324 | 6353 | 0.007216661 | 0.716495409 |
| 60 minutes | 010517_T3_chain_15.nrrd | Synech_2 | 588844 | 5436 | 0.009231647 | 0.914720334 |
| 60 minutes | 010517_T3_chain_15.nrrd | Synech_3 | 594284 | 5307 | 0.008930074 | 0.885103345 |
| 60 minutes | 010517_T3_chain_15.nrrd | Synech_4 | 786738 | 5947 | 0.00755906 | 0.750234961 |
| 60 minutes | 010517_T3_chain_16.nrrd | Synech_1 | 387806 | 3148 | 0.008117461 | 0.80520982 |
| 60 minutes | 010517_T3_chain_16.nrrd | Synech_10 | 587485 | 3663 | 0.006235053 | 0.619641782 |
| 60 minutes | 010517_T3_chain_16.nrrd | Synech_11 | 538773 | 2073 | 0.003847632 | 0.383288404 |
| 60 minutes | 010517_T3_chain_16.nrrd | Synech_2 | 496883 | 2135 | 0.004296786 | 0.427840278 |
| 60 minutes | 010517_T3_chain_16.nrrd | Synech_3 | 377297 | 2576 | 0.006827513 | 0.678121372 |
| 60 minutes | 010517_T3_chain_16.nrrd | Synech_4 | 418248 | 2670 | 0.006383772 | 0.634327826 |
| 60 minutes | 010517_T3_chain_16.nrrd | Synech_5 | 411044 | 5532 | 0.013458413 | 1.327968966 |
| 60 minutes | 010517_T3_chain_16.nrrd | Synech_6 | 321094 | 2981 | 0.009283886 | 0.9198488 |
| 60 minutes | 010517_T3_chain_16.nrrd | Synech_7 | 364491 | 1748 | 0.004795729 | 0.477283959 |
| 60 minutes | 010517_T3_chain_16.nrrd | Synech_8 | 290464 | 2028 | 0.006981932 | 0.693352297 |
| 120 minutes | 010517_T4_12.nrrd | Synech_1 | 494297 | 2912 | 0.005891195 | 0.585669206 |
| 120 minutes | 010517_T4_12.nrrd | Synech_2 | 405868 | 8314 | 0.020484492 | 2.007330111 |
| 120 minutes | 010517_T4_12.nrrd | Synech_3 | 373145 | 5242 | 0.014048158 | 1.385354148 |
| 120 minutes | 010517_T4_12.nrrd | Synech_4 | 439679 | 11104 | 0.025254788 | 2.463269467 |
| 120 minutes | 010517_T4_12.nrrd | Synech_5 | 179190 | 3805 | 0.021234444 | 2.079291784 |
| 120 minutes | 010517_T4_12.nrrd | Synech_6 | 263354 | 6175 | 0.023447527 | 2.291033618 |
| 120 minutes | 010517_T4_12.nrrd | Synech_7 | 206608 | 4022 | 0.019466816 | 1.909509567 |
| 120 minutes | 010517_T4_12.nrrd | Synech_8 | 303522 | 11048 | 0.036399338 | 3.512095877 |
| 120 minutes | 010517_T4_12.nrrd | Synech_9 | 254601 | 8902 | 0.034964513 | 3.378329658 |
| 120 minutes | 010517_T4_13.nrrd | Synech_1 | 459626 | 11146 | 0.024250151 | 2.367600452 |
| 120 minutes | 010517_T4_13.nrrd | Synech_2 | 390703 | 4645 | 0.011888826 | 1.174914253 |
| 120 minutes | 010517_T4_13.nrrd | Synech_3 | 290872 | 5249 | 0.018045738 | 1.772586206 |
| 120 minutes | 010517_T4_13.nrrd | Synech_4 | 232907 | 6172 | 0.026499848 | 2.581573455 |
| 120 minutes | 010517_T4_13.nrrd | Synech_5 | 359197 | 3778 | 0.010517905 | 1.040843033 |
| 120 minutes | 010517_T4_13.nrrd | Synech_6 | 385488 | 8795 | 0.022815237 | 2.230631298 |
| 120 minutes | 010517_T4_13.nrrd | Synech_7 | 131667 | 2465 | 0.018721472 | 1.837741926 |
| 120 minutes | 010517_T4_13.nrrd | Synech_8 | 167645 | 2820 | 0.016821259 | 1.654298536 |
| 120 minutes | 010517_T4_14.nrrd | Synech_1 | 563294 | 16932 | 0.030058904 | 2.918173264 |
| 120 minutes | 010517_T4_14.nrrd | Synech_2 | 389390 | 6039 | 0.015508873 | 1.527202102 |
| 120 minutes | 010517_T4_14.nrrd | Synech_3 | 524523 | 12828 | 0.024456506 | 2.387266424 |
| 120 minutes | 010517_T4_14.nrrd | Synech_4 | 419759 | 5446 | 0.012974111 | 1.28079397 |
| 120 minutes | 010517_T4_14.nrrd | Synech_5 | 561962 | 13643 | 0.024277442 | 2.370201788 |
| 120 minutes | 010517_T4_chain_1.nrrd | Synech_1 | 500632 | 8484 | 0.01694658 | 1.666417869 |
| 120 minutes | 010517_T4_chain_1.nrrd | Synech_2 | 531357 | 21209 | 0.039914784 | 3.838274523 |
| 120 minutes | 010517_T4_chain_2.nrrd | Synech_1 | 464334 | 5320 | 0.01145727 | 1.13274879 |
| 120 minutes | 010517_T4_chain_2.nrrd | Synech_2 | 533195 | 8986 | 0.016853121 | 1.6573801 |
| 120 minutes | 010517_T4_chain_3.nrrd | Synech_1 | 465735 | 4887 | 0.010493092 | 1.038412994 |
| 120 minutes | 010517_T4_chain_3.nrrd | Synech_2 | 448831 | 13009 | 0.028984183 | 2.816776373 |
| 120 minutes | 010517_T4_chain_3.nrrd | Synech_3 | 421665 | 7171 | 0.017006391 | 1.672201028 |
| 120 minutes | 010517_T4_chain_4.nrrd | Synech_1 | 516431 | 12019 | 0.023273196 | 2.274387359 |
| 120 minutes | 010517_T4_chain_4.nrrd | Synech_2 | 404146 | 4413 | 0.010919321 | 1.080137752 |
| 120 minutes | 010517_T4_chain_5.nrrd | Synech_1 | 365230 | 8759 | 0.023982148 | 2.342047493 |
| 120 minutes | 010517_T4_chain_5.nrrd | Synech_2 | 317891 | 3175 | 0.0099877 | 0.988893249 |
| 120 minutes | 010517_T4_chain_6.nrrd | Synech_1 | 756632 | 14901 | 0.019693854 | 1.931349664 |
| 120 minutes | 010517_T4_chain_6.nrrd | Synech_2 | 438698 | 9613 | 0.021912569 | 2.144270384 |
| 120 minutes | 010517_T4_chain_7.nrrd | Synech_1 | 428461 | 14512 | 0.033870061 | 3.27604617 |
| 120 minutes | 010517_T4_chain_7.nrrd | Synech_2 | 542866 | 13247 | 0.02440197 | 2.382069831 |
| 120 minutes | 010517_T4_chain_7.nrrd | Synech_3 | 657271 | 11982 | 0.018229923 | 1.79035432 |
| 120 minutes | 010517_T4_chain_8.nrrd | Synech_1 | 472101 | 8295 | 0.017570393 | 1.726700472 |
| 120 minutes | 010517_T4_chain_8.nrrd | Synech_2 | 541282 | 7295 | 0.013477263 | 1.329804203 |
| 120 minutes | 010517_T4_chain_9.nrrd | Synech_1 | 465300 | 5133 | 0.011031593 | 1.091122434 |
| 120 minutes | 010517_T4_chain_9.nrrd | Synech_2 | 437983 | 4821 | 0.011007277 | 1.088743552 |
| 120 minutes | 010517_T4_chain_9.nrrd | Synech_3 | 365440 | 12806 | 0.035042688 | 3.385627343 |
| 120 minutes | 010517_T4_chain_9.nrrd | Synech_4 | 339580 | 3544 | 0.010436421 | 1.032862755 |
| 120 minutes | 010517_T4_chain_9.nrrd | Synech_5 | 309944 | 5427 | 0.017509615 | 1.720830387 |
| 120 minutes | 010517_T4_chain_10.nrrd | Synech_1 | 652244 | 13494 | 0.020688577 | 2.026923504 |
| 120 minutes | 010517_T4_chain_10.nrrd | Synech_2 | 578631 | 9975 | 0.017238966 | 1.694682011 |
| 120 minutes | 010517_T4_chain_10.nrrd | Synech_3 | 488751 | 10116 | 0.020697656 | 2.027794983 |
| 120 minutes | 010517_T4_chain_11.nrrd | Synech_1 | 405698 | 8575 | 0.021136412 | 2.06989111 |
| 120 minutes | 010517_T4_chain_11.nrrd | Synech_2 | 605029 | 27453 | 0.045374685 | 4.340518782 |
| 120 minutes | 010517_T4_chain_11.nrrd | Synech_3 | 351481 | 8943 | 0.025443765 | 2.481244312 |
| 120 minutes | 010517_T4_chain_12.nrrd | Synech_1 | 642843 | 11104 | 0.017273269 | 1.697996932 |
| 120 minutes | 010517_T4_chain_12.nrrd | Synech_2 | 664954 | 15416 | 0.023183559 | 2.265825948 |
| 120 minutes | 010517_T4_chain_13.nrrd | Synech_1 | 604199 | 9905 | 0.016393605 | 1.612918984 |
| 120 minutes | 010517_T4_chain_13.nrrd | Synech_2 | 432525 | 7436 | 0.01719207 | 1.690149809 |
| 120 minutes | 010517_T4_chain_14.nrrd | Synech_1 | 791552 | 11062 | 0.013975077 | 1.378246579 |
| 120 minutes | 010517_T4_chain_14.nrrd | Synech_2 | 441138 | 8367 | 0.018966854 | 1.861380852 |
| 120 minutes | 010517_T4_chain_15.nrrd | Synech_1 | 366681 | 6666 | 0.018179289 | 1.785470353 |
| 120 minutes | 010517_T4_chain_15.nrrd | Synech_2 | 475744 | 5267 | 0.01107108 | 1.094985354 |
| 240 minutes | 260417_T5_chain_1.nrrd | Synech_1 | 829095 | 42530 | 0.051296896 | 4.87939194 |
| 240 minutes | 260417_T5_chain_1.nrrd | Synech_2 | 532935 | 49764 | 0.093377241 | 8.54025835 |
| 240 minutes | 260417_T5_chain_2.nrrd | Synech_1 | 818011 | 4019 | 0.004913137 | 0.488911597 |
| 240 minutes | 260417_T5_chain_2.nrrd | Synech_2 | 589301 | 14704 | 0.024951595 | 2.434416934 |
| 240 minutes | 260417_T5_chain_3.nrrd | Synech_1 | 676314 | 44249 | 0.06542671 | 6.140892608 |
| 240 minutes | 260417_T5_chain_4.nrrd | Synech_1 | 651228 | 32987 | 0.050653535 | 4.8211454 |
| 240 minutes | 260417_T5_chain_4.nrrd | Synech_2 | 634132 | 27443 | 0.043276479 | 4.148131353 |
| 240 minutes | 260417_T5_chain_4.nrrd | Synech_3 | 517232 | 18230 | 0.035245306 | 3.404536643 |
| 240 minutes | 260417_T5_chain_4.nrrd | Synech_4 | 581876 | 57142 | 0.098203054 | 8.942158124 |
| 240 minutes | 260417_T5_chain_4.nrrd | Synech_5 | 475297 | 44113 | 0.092811442 | 8.492905412 |
| 240 minutes | 260417_T5_chain_5.nrrd | Synech_1 | 734557 | 34144 | 0.046482438 | 4.441779053 |
| 240 minutes | 260417_T5_chain_5.nrrd | Synech_2 | 619709 | 45569 | 0.0735329 | 6.849617754 |
| 240 minutes | 260417_T5_chain_5.nrrd | Synech_3 | 431798 | 24690 | 0.057179514 | 5.408685442 |
| 240 minutes | 260417_T5_chain_5.nrrd | Synech_4 | 542244 | 25856 | 0.047683331 | 4.551311389 |
| 240 minutes | 260417_T5_chain_5.nrrd | Synech_5 | 376229 | 19911 | 0.052922555 | 5.026253345 |
| 240 minutes | 260417_T5_chain_5.nrrd | Synech_6 | 484460 | 25761 | 0.053174669 | 5.048988576 |
| 240 minutes | 260417_T5_chain_6.nrrd | Synech_1 | 485883 | 42361 | 0.08718354 | 8.019210819 |
| 240 minutes | 260417_T5_chain_6.nrrd | Synech_2 | 802326 | 50352 | 0.062757532 | 5.905159978 |
| 240 minutes | 260417_T5_chain_7.nrrd | Synech_1 | 505036 | 28557 | 0.056544484 | 5.351831827 |
| 240 minutes | 260417_T5_chain_7.nrrd | Synech_2 | 756236 | 53933 | 0.071317684 | 6.657006131 |
| 240 minutes | 260417_T5_chain_7.nrrd | Synech_3 | 687915 | 26570 | 0.038623958 | 3.718762465 |
| 240 minutes | 260417_T5_chain_8.nrrd | Synech_1 | 767143 | 46767 | 0.060962558 | 5.745966999 |
| 240 minutes | 260417_T5_chain_8.nrrd | Synech_2 | 715412 | 30456 | 0.042571274 | 4.08329624 |
| 240 minutes | 260417_T5_chain_8.nrrd | Synech_3 | 1156179 | 51510 | 0.044551925 | 4.265170917 |
| 240 minutes | 260417_T5_chain_9.nrrd | Synech_1 | 885333 | 39668 | 0.04480574 | 4.288427796 |
| 240 minutes | 260417_T5_chain_9.nrrd | Synech_2 | 439405 | 34031 | 0.077447913 | 7.188088781 |
| 240 minutes | 260417_T5_chain_9.nrrd | Synech_3 | 760358 | 50329 | 0.066191189 | 6.208191324 |
| 240 minutes | 260417_T5_chain_10.nrrd | Synech_1 | 802221 | 61109 | 0.07617477 | 7.078289878 |
| 240 minutes | 260417_T5_chain_10.nrrd | Synech_2 | 521223 | 32633 | 0.062608519 | 5.891964698 |
| 240 minutes | 260417_T5_chain_10.nrrd | Synech_3 | 582663 | 36153 | 0.062047873 | 5.842285914 |
| 240 minutes | 260417_T5_chain_11.nrrd | Synech_1 | 451870 | 6388 | 0.014136809 | 1.393974573 |
| 240 minutes | 260417_T5_chain_11.nrrd | Synech_2 | 396045 | 1818 | 0.004590387 | 0.456941208 |
| 240 minutes | 260417_T5_chain_12.nrrd | Synech_1 | 865922 | 56911 | 0.06572301 | 6.16698796 |
| 240 minutes | 260417_T5_chain_12.nrrd | Synech_2 | 526677 | 43937 | 0.083423047 | 7.699951281 |
| 240 minutes | 260417_T5_chain_13.nrrd | Synech_1 | 550824 | 22873 | 0.041525061 | 3.986947814 |
| 240 minutes | 260417_T5_chain_13.nrrd | Synech_2 | 755710 | 25364 | 0.033563139 | 3.247323557 |
| 240 minutes | 260417_T5_chain_13.nrrd | Synech_3 | 717170 | 37358 | 0.052090857 | 4.951174774 |
| 240 minutes | 260417_T5_chain_13.nrrd | Synech_4 | 818934 | 52839 | 0.064521683 | 6.06109618 |
| 240 minutes | 260417_T5_chain_14.nrrd | Synech_1 | 494011 | 34192 | 0.069213034 | 6.4732688 |
| 240 minutes | 260417_T5_chain_14.nrrd | Synech_2 | 837820 | 38750 | 0.046250985 | 4.420639538 |
| 240 minutes | 260417_T5_chain_15.nrrd | Synech_1 | 680662 | 52423 | 0.077017668 | 7.151012502 |
| 240 minutes | 260417_T5_chain_15.nrrd | Synech_2 | 694523 | 34963 | 0.050341025 | 4.79282673 |
| 240 minutes | 260417_T5_chain_15.nrrd | Synech_3 | 746404 | 44011 | 0.058964046 | 5.56808765 |
| 240 minutes | 260417_T5_chain_15.nrrd | Synech_4 | 413576 | 14051 | 0.033974409 | 3.285807491 |
| 240 minutes | 260417_T5_chain_16.nrrd | Synech_1 | 534787 | 46228 | 0.086441892 | 7.956421091 |
| 240 minutes | 260417_T5_chain_16.nrrd | Synech_2 | 594521 | 36958 | 0.062164331 | 5.852609509 |
| 240 minutes | 260417_T5_chain_17.nrrd | Synech_1 | 497609 | 32264 | 0.064838056 | 6.089006234 |
| 240 minutes | 260417_T5_chain_18.nrrd | Synech_1 | 632426 | 15196 | 0.024028108 | 2.34643048 |
| 240 minutes | 260417_T5_chain_18.nrrd | Synech_2 | 546295 | 12315 | 0.022542765 | 2.204579223 |
| 240 minutes | 260417_T5_chain_18.nrrd | Synech_3 | 766176 | 43143 | 0.056309516 | 5.330778099 |
| 240 minutes | 260417_T5_chain_18.nrrd | Synech_4 | 623900 | 53350 | 0.085510498 | 7.877445552 |
| 240 minutes | 260417_T5_chain_18.nrrd | Synech_5 | 681732 | 41823 | 0.061348154 | 5.780210212 |
| 240 minutes | 260417_T5_chain_18.nrrd | Synech_6 | 468861 | 19427 | 0.041434455 | 3.9785946 |
| 360 minutes | 020517_T6_chain_1.nrrd | Synech_1 | 746785 | 100096 | 0.134035901 | 11.81937014 |
| 360 minutes | 020517_T6_chain_1.nrrd | Synech_2 | 594592 | 68897 | 0.115872733 | 10.38404555 |
| 360 minutes | 020517_T6_chain_1.nrrd | Synech_3 | 444069 | 47517 | 0.107003641 | 9.666060466 |
| 360 minutes | 020517_T6_chain_1.nrrd | Synech_4 | 595772 | 41430 | 0.069540025 | 6.501862832 |
| 360 minutes | 020517_T6_chain_1.nrrd | Synech_5 | 619513 | 100759 | 0.162642269 | 13.98902081 |
| 360 minutes | 020517_T6_chain_1.nrrd | Synech_6 | 590604 | 98138 | 0.166165485 | 14.24887694 |
| 360 minutes | 020517_T6_chain_1.nrrd | Synech_7 | 440701 | 57785 | 0.131120646 | 11.59210088 |
| 360 minutes | 020517_T6_chain_2.nrrd | Synech_1 | 493545 | 60440 | 0.122460971 | 10.91004269 |
| 360 minutes | 020517_T6_chain_2.nrrd | Synech_2 | 590728 | 35741 | 0.060503311 | 5.705150614 |
| 360 minutes | 020517_T6_chain_2.nrrd | Synech_3 | 502157 | 30714 | 0.061164138 | 5.763871556 |
| 360 minutes | 020517_T6_chain_3.nrrd | Synech_1 | 569818 | 55199 | 0.096871282 | 8.83159978 |
| 360 minutes | 020517_T6_chain_3.nrrd | Synech_2 | 599920 | 66623 | 0.11105314 | 9.995304129 |
| 360 minutes | 020517_T6_chain_3.nrrd | Synech_3 | 654075 | 4797 | 0.007334021 | 0.728062507 |
| 360 minutes | 020517_T6_chain_3.nrrd | Synech_4 | 707596 | 88081 | 0.124479223 | 11.06994421 |
| 360 minutes | 020517_T6_chain_3.nrrd | Synech_5 | 913891 | 61993 | 0.067834129 | 6.352496813 |
| 360 minutes | 020517_T6_chain_3.nrrd | Synech_6 | 423774 | 42558 | 0.100426171 | 9.126116158 |
| 360 minutes | 020517_T6_chain_3.nrrd | Synech_7 | 513050 | 60311 | 0.117553845 | 10.51885287 |
| 360 minutes | 020517_T6_chain_3.nrrd | Synech_8 | 760855 | 15525 | 0.020404676 | 1.999665112 |
| 360 minutes | 020517_T6_chain_4.nrrd | Synech_1 | 750449 | 80976 | 0.107903402 | 9.739423279 |
| 360 minutes | 020517_T6_chain_4.nrrd | Synech_2 | 603189 | 84486 | 0.140065552 | 12.28574545 |
| 360 minutes | 020517_T6_chain_5.nrrd | Synech_1 | 485278 | 50605 | 0.104280433 | 9.443292659 |
| 360 minutes | 020517_T6_chain_5.nrrd | Synech_2 | 414504 | 38041 | 0.091774748 | 8.406014871 |
| 360 minutes | 020517_T6_chain_5.nrrd | Synech_3 | 352774 | 44554 | 0.126296156 | 11.21340555 |
| 360 minutes | 020517_T6_chain_5.nrrd | Synech_4 | 371393 | 48090 | 0.129485478 | 11.46411178 |
| 360 minutes | 020517_T6_chain_6.nrrd | Synech_1 | 717389 | 83529 | 0.116434738 | 10.42915754 |
| 360 minutes | 020517_T6_chain_6.nrrd | Synech_2 | 876056 | 88095 | 0.10055864 | 9.137054258 |
| 360 minutes | 020517_T6_chain_6.nrrd | Synech_3 | 490549 | 53825 | 0.109724003 | 9.887503812 |
| 360 minutes | 020517_T6_chain_6.nrrd | Synech_4 | 422366 | 67531 | 0.159887396 | 13.78473434 |
| 360 minutes | 020517_T6_chain_7.nrrd | Synech_1 | 660704 | 103692 | 0.156941686 | 13.56522012 |
| 360 minutes | 020517_T6_chain_7.nrrd | Synech_2 | 650957 | 69126 | 0.106191346 | 9.599726698 |
| 360 minutes | 020517_T6_chain_8.nrrd | Synech_1 | 562780 | 60214 | 0.106993852 | 9.665261624 |
| 360 minutes | 020517_T6_chain_8.nrrd | Synech_2 | 489564 | 62739 | 0.128152805 | 11.35952548 |
| 360 minutes | 020517_T6_chain_8.nrrd | Synech_3 | 466660 | 46588 | 0.099832855 | 9.077093335 |
| 360 minutes | 020517_T6_chain_8.nrrd | Synech_4 | 494248 | 57542 | 0.116423334 | 10.42824263 |
| 360 minutes | 020517_T6_chain_8.nrrd | Synech_5 | 462017 | 59129 | 0.127980139 | 11.3459568 |
| 360 minutes | 020517_T6_chain_8.nrrd | Synech_6 | 868879 | 52817 | 0.06078752 | 5.730414367 |
| 360 minutes | 020517_T6_chain_8.nrrd | Synech_7 | 364831 | 38097 | 0.104423692 | 9.455039114 |
| 360 minutes | 020517_T6_chain_8.nrrd | Synech_8 | 353029 | 37674 | 0.106716445 | 9.642618562 |
| 360 minutes | 020517_T6_chain_9.nrrd | Synech_1 | 716296 | 104978 | 0.146556731 | 12.78233574 |
| 360 minutes | 020517_T6_chain_9.nrrd | Synech_2 | 710006 | 92652 | 0.130494672 | 11.54314789 |
| 360 minutes | 020517_T6_chain_9.nrrd | Synech_3 | 392583 | 42911 | 0.109304275 | 9.853407854 |
| 360 minutes | 020517_T6_chain_10.nrrd | Synech_1 | 638051 | 3287 | 0.005151626 | 0.512522258 |
| 360 minutes | 020517_T6_chain_10.nrrd | Synech_2 | 557552 | 74069 | 0.132846802 | 11.72681086 |
| 360 minutes | 020517_T6_chain_11.nrrd | Synech_1 | 635193 | 114560 | 0.180354632 | 15.27969878 |
| 360 minutes | 020517_T6_chain_11.nrrd | Synech_2 | 607590 | 87843 | 0.144576112 | 12.63141093 |
| 360 minutes | 020517_T6_chain_12.nrrd | Synech_1 | 571197 | 72746 | 0.127357111 | 11.29696262 |
| 360 minutes | 020517_T6_chain_12.nrrd | Synech_2 | 636322 | 4081 | 0.00641342 | 0.637254979 |
| 360 minutes | 020517_T6_chain_12.nrrd | Synech_3 | 448547 | 36283 | 0.080890074 | 7.483654064 |
| 360 minutes | 020517_T6_chain_13.nrrd | Synech_1 | 772028 | 102500 | 0.132767205 | 11.72060815 |
| 360 minutes | 020517_T6_chain_13.nrrd | Synech_2 | 699461 | 42476 | 0.06072676 | 5.725014388 |
| 360 minutes | 020517_T6_chain_13.nrrd | Synech_3 | 392724 | 35047 | 0.08924079 | 8.192935005 |
| 360 minutes | 020517_T6_chain_14.nrrd | Synech_1 | 624513 | 22621 | 0.036221824 | 3.495566606 |
| 360 minutes | 020517_T6_chain_14.nrrd | Synech_2 | 416044 | 41964 | 0.100864332 | 9.162285375 |
| 360 minutes | 020517_T6_chain_15.nrrd | Synech_1 | 512029 | 56242 | 0.109841435 | 9.897038561 |
| 360 minutes | 020517_T6_chain_15.nrrd | Synech_2 | 458259 | 40386 | 0.088129202 | 8.099148693 |
| 360 minutes | 020517_T6_chain_15.nrrd | Synech_3 | 645814 | 45193 | 0.069978353 | 6.540165295 |
| 360 minutes | 020517_T6_chain_16.nrrd | Synech_1 | 506829 | 54542 | 0.107614205 | 9.715856359 |
| 360 minutes | 020517_T6_chain_16.nrrd | Synech_2 | 468016 | 50505 | 0.107912977 | 9.740203386 |
| 360 minutes | 020517_T6_chain_16.nrrd | Synech_3 | 403359 | 46905 | 0.116285988 | 10.4172219 |
| 360 minutes | 020517_T6_chain_16.nrrd | Synech_4 | 573161 | 69686 | 0.121581894 | 10.84021548 |
| 360 minutes | 020517_T6_chain_17.nrrd | Synech_1 | 769941 | 62204 | 0.080790606 | 7.475139549 |
| 360 minutes | 020517_T6_chain_17.nrrd | Synech_2 | 544149 | 73166 | 0.134459495 | 11.85229583 |
| 360 minutes | 020517_T6_chain_18.nrrd | Synech_1 | 381180 | 51260 | 0.13447715 | 11.85366756 |
| 360 minutes | 020517_T6_chain_18.nrrd | Synech_2 | 389010 | 46153 | 0.118642194 | 10.60591089 |
| 360 minutes | 020517_T6_chain_18.nrrd | Synech_3 | 411690 | 44805 | 0.108831888 | 9.81500345 |
| 360 minutes | 020517_T6_chain_18.nrrd | Synech_4 | 530035 | 45738 | 0.086292415 | 7.943755612 |
| 360 minutes | 020517_T6_chain_18.nrrd | Synech_5 | 534687 | 11532 | 0.021567758 | 2.111241096 |
| 360 minutes | 020517_T6_chain_18.nrrd | Synech_6 | 423742 | 29564 | 0.069768869 | 6.521863818 |
| 360 minutes | 020517_T6_chain_18.nrrd | Synech_7 | 416175 | 28753 | 0.069088725 | 6.462393915 |
| 360 minutes | 020517_T6_chain_18.nrrd | Synech_8 | 309386 | 29670 | 0.095899621 | 8.750766835 |
| 360 minutes | 020517_T6_chain_18.nrrd | Synech_9 | 247585 | 24683 | 0.099695054 | 9.065699972 |
|  |  |  |  |  |  |  |

**Supplementary Table 4.** Total nitrogen quantification with ToF-SIMS.

| Time | File name | ROI name | ^12^C^14^N counts | ^12^C^15^N counts | Ratio | AT% |
| --- | --- | --- | --- | --- | --- | --- |
| Negative control | neg-ArDP1-Bi3-Ar10keV_100-128-r340_neg2 | Roi 2 | 141370.7 | 633.7 | 0.00448254 | 0.44625378 |
| Negative control | neg-ArDP1-Bi3-Ar10keV_100-128-r340_neg2 | Roi 3 | 17832.7 | 101.4 | 0.00568618 | 0.56540334 |
| Negative control | neg-ArDP1-Bi3-Ar10keV_100-128-r340_neg2 | Roi 4 | 65756 | 295 | 0.00448628 | 0.44662458 |
| Negative control | neg-ArDP1-Bi3-Ar10keV_100-128-r340_neg2 | Roi 5 | 165262.7 | 869.9 | 0.00526374 | 0.52361788 |
| Negative control | neg-ArDP1-Bi3-Ar10keV_100-128-r340_neg2 | Roi 6 | 56602.8 | 284.4 | 0.00502449 | 0.49993672 |
| Negative control | neg-ArDP1-Bi3-Ar10keV_100-128-r340_neg2 | Roi 7 | 109308.4 | 583.4 | 0.00533719 | 0.53088583 |
| Negative control | neg-ArDP1-Bi3-Ar10keV_100-128-r340_neg2 | Roi 8 | 34004.2 | 184.7 | 0.00543168 | 0.54023382 |
| Negative control | neg-ArDP1-Bi3-Ar10keV_100-128-r340_neg2 | Roi 9 | 32941.4 | 149 | 0.00452318 | 0.45028165 |
| Negative control | neg-ArDP1-Bi3-Ar10keV_100-128-r340_neg2 | Roi 10 | 22408.6 | 98.2 | 0.00438225 | 0.43631258 |
| Negative control | neg-ArDP2-Bi3-Ar10keV_100-128-r340_neg5 | Roi 2 | 83069.2 | 348.7 | 0.00419771 | 0.4180158 |
| Negative control | neg-ArDP2-Bi3-Ar10keV_100-128-r340_neg5 | Roi 3 | 56042 | 222.5 | 0.00397024 | 0.39545362 |
| Negative control | neg-ArDP2-Bi3-Ar10keV_100-128-r340_neg5 | Roi 4 | 41103.8 | 208.4 | 0.00507009 | 0.50445147 |
| Negative control | neg-ArDP2-Bi3-Ar10keV_100-128-r340_neg5 | Roi 5 | 309893.4 | 1266.3 | 0.00408624 | 0.40696144 |
| Negative control | neg-ArDP2-Bi3-Ar10keV_100-128-r340_neg5 | Roi 6 | 16105.7 | 99.3 | 0.00616552 | 0.61277384 |
| Negative control | neg-ArDP2-Bi3-Ar10keV_100-128-r340_neg5 | Roi 7 | 58808.4 | 255 | 0.00433612 | 0.43173945 |
| Negative control | neg-ArDP3-Bi3-Ar10keV_100-128-r340_neg6 | Roi 2 | 6702.8 | 24.2 | 0.00361043 | 0.35974431 |
| Negative control | neg-ArDP3-Bi3-Ar10keV_100-128-r340_neg6 | Roi 3 | 15682.3 | 58.1 | 0.00370481 | 0.36911387 |
| Negative control | neg-ArDP3-Bi3-Ar10keV_100-128-r340_neg6 | Roi 4 | 22324.9 | 98.4 | 0.00440763 | 0.43882925 |
| Negative control | neg-ArDP3-Bi3-Ar10keV_100-128-r340_neg6 | Roi 5 | 19154.9 | 109.4 | 0.00571133 | 0.56788983 |
| Negative control | neg-ArDP3-Bi3-Ar10keV_100-128-r340_neg6 | Roi 6 | 45752.6 | 185.3 | 0.00405004 | 0.40337064 |
| Negative control | neg-ArDP3-Bi3-Ar10keV_100-128-r340_neg6 | Roi 7 | 33719.6 | 130 | 0.00385532 | 0.38405181 |
| Negative control | neg-DP1_Bi3-Ar20keV_146-256-r340_neg2 | Roi 1 | 27909 | 150.7 | 0.00539969 | 0.53706918 |
| Negative control | neg-DP1_Bi3-Ar20keV_146-256-r340_neg2 | Roi 2 | 17337.8 | 55.3 | 0.00318956 | 0.31794217 |
| 15 minutes | T1-ArDP1-Bi3-Ar10keV_100-128-r340_neg55 | Roi 2 | 41332.9 | 222.6 | 0.00538554 | 0.53566917 |
| 15 minutes | T1-ArDP1-Bi3-Ar10keV_100-128-r340_neg55 | Roi 3 | 27618.4 | 161 | 0.00582945 | 0.57956615 |
| 15 minutes | T1-ArDP1-Bi3-Ar10keV_100-128-r340_neg55 | Roi 4 | 44397.3 | 271.6 | 0.00611749 | 0.6080293 |
| 15 minutes | T1-ArDP1-Bi3-Ar10keV_100-128-r340_neg55 | Roi 5 | 20151.9 | 117.4 | 0.00582575 | 0.57920106 |
| 15 minutes | T1-ArDP1-Bi3-Ar10keV_100-128-r340_neg55 | Roi 6 | 40796.1 | 217.4 | 0.00532894 | 0.53006937 |
| 15 minutes | T1-ArDP1-Bi3-Ar10keV_100-128-r340_neg55 | Roi 7 | 17047.5 | 102.9 | 0.00603608 | 0.59998601 |
| 15 minutes | T1-ArDP1-Bi3-Ar10keV_100-128-r340_neg55 | Roi 8 | 42702.3 | 225 | 0.00526904 | 0.52414198 |
| 15 minutes | T1-ArDP2-Bi3-Ar10keV_100-128-r340_neg56 | Roi 2 | 11562.3 | 81 | 0.00700553 | 0.69567906 |
| 15 minutes | T1-ArDP2-Bi3-Ar10keV_100-128-r340_neg56 | Roi 3 | 19207 | 85 | 0.00442547 | 0.44059714 |
| 15 minutes | T1-ArDP2-Bi3-Ar10keV_100-128-r340_neg56 | Roi 4 | 45331.1 | 225.8 | 0.00498113 | 0.49564391 |
| 15 minutes | T1-ArDP2-Bi3-Ar10keV_100-128-r340_neg56 | Roi 5 | 22434.5 | 106.3 | 0.00473824 | 0.4715893 |
| 15 minutes | T1-ArDP2-Bi3-Ar10keV_100-128-r340_neg56 | Roi 6 | 8206.4 | 52.9 | 0.00644619 | 0.64049011 |
| 15 minutes | T1-ArDP2-Bi3-Ar10keV_100-128-r340_neg56 | Roi 7 | 30848.1 | 165.1 | 0.00535203 | 0.53235397 |
| 15 minutes | T1-ArDP2-Bi3-Ar10keV_100-128-r340_neg56 | Roi 9 | 67278.8 | 344.2 | 0.00511602 | 0.50899842 |
| 15 minutes | T1-ArDP2-Bi3-Ar10keV_100-128-r340_neg56 | Roi 11 | 3775 | 24.4 | 0.00646358 | 0.64220666 |
| 15 minutes | T1-ArDP2-Bi3-Ar10keV_100-128-r340_neg56 | Roi 12 | 5858.5 | 36.7 | 0.0062644 | 0.62254037 |
| 15 minutes | T1-ArDP2-Bi3-Ar10keV_100-128-r340_neg56 | Roi 13 | 17419.2 | 104.7 | 0.00601061 | 0.59746974 |
| 15 minutes | T1-ArDP3-Bi3-Ar10keV_100-128-r340_neg57 | Roi 2 | 148888.1 | 774.5 | 0.00520189 | 0.51749736 |
| 15 minutes | T1-ArDP3-Bi3-Ar10keV_100-128-r340_neg57 | Roi 3 | 90795.6 | 449.9 | 0.00495509 | 0.49306541 |
| 15 minutes | T1-ArDP3-Bi3-Ar10keV_100-128-r340_neg57 | Roi 4 | 99626.9 | 466.7 | 0.00468448 | 0.46626358 |
| 15 minutes | T1-ArDP3-Bi3-Ar10keV_100-128-r340_neg57 | Roi 5 | 44382.5 | 260.2 | 0.00586267 | 0.58285005 |
| 15 minutes | T1-ArDP3-Bi3-Ar10keV_100-128-r340_neg57 | Roi 6 | 9099.7 | 35.8 | 0.0039342 | 0.39187784 |
| 15 minutes | T1-ArDP3-Bi3-Ar10keV_100-128-r340_neg57 | Roi 7 | 5682.5 | 17.1 | 0.00300924 | 0.30002105 |
| 15 minutes | T1-ArDP3-Bi3-Ar10keV_100-128-r340_neg57 | Roi 8 | 9197.2 | 55.8 | 0.00606706 | 0.60304766 |
| 30 minutes | T2-ArDP1-Bi3-Ar10keV_100-128-r340_neg53 | Roi 2 | 24366.3 | 246.3 | 0.010108223 | 1.000706955 |
| 30 minutes | T2-ArDP1-Bi3-Ar10keV_100-128-r340_neg53 | Roi 3 | 26950.6 | 179.5 | 0.006660334 | 0.661626754 |
| 30 minutes | T2-ArDP1-Bi3-Ar10keV_100-128-r340_neg53 | Roi 4 | 9070.4 | 43.5 | 0.004795819 | 0.477292926 |
| 30 minutes | T2-ArDP1-Bi3-Ar10keV_100-128-r340_neg53 | Roi 5 | 111742.8 | 1140 | 0.010202 | 1.009896991 |
| 30 minutes | T2-ArDP1-Bi3-Ar10keV_100-128-r340_neg53 | Roi 6 | 60205.2 | 476.1 | 0.007907955 | 0.78459097 |
| 30 minutes | T2-ArDP1-Bi3-Ar10keV_100-128-r340_neg53 | Roi 7 | 33468.3 | 239.2 | 0.007147062 | 0.709634354 |
| 30 minutes | T2-ArDP1-Bi3-Ar10keV_100-128-r340_neg53 | Roi 8 | 28013.6 | 185.1 | 0.006607505 | 0.656413239 |
| 30 minutes | T2-ArDP1-Bi3-Ar10keV_100-128-r340_neg53 | Roi 9 | 39412.2 | 236.8 | 0.006008292 | 0.597240788 |
| 30 minutes | T2-ArDP2-Bi3-Ar10keV_100-128-r340_neg54 | Roi 1 | 544696.5 | 4021.5 | 0.007383011 | 0.732890118 |
| 30 minutes | T2-ArDP2-Bi3-Ar10keV_100-128-r340_neg54 | Roi 2 | 66814.1 | 505.4 | 0.007564272 | 0.750748297 |
| 30 minutes | T2-ArDP2-Bi3-Ar10keV_100-128-r340_neg54 | Roi 3 | 102854 | 677 | 0.006582146 | 0.653910423 |
| 30 minutes | T2-ArDP2-Bi3-Ar10keV_100-128-r340_neg54 | Roi 4 | 121026.6 | 931.2 | 0.007694176 | 0.763542799 |
| 30 minutes | T2-ArDP2-Bi3-Ar10keV_100-128-r340_neg54 | Roi 5 | 40675.2 | 288.2 | 0.007085398 | 0.703554881 |
| 30 minutes | T2-ArDP2-Bi3-Ar10keV_100-128-r340_neg54 | Roi 6 | 8251.9 | 38.2 | 0.004629237 | 0.460790582 |
| 30 minutes | T2-ArDP2-Bi3-Ar10keV_100-128-r340_neg54 | Roi 7 | 47517.7 | 426.5 | 0.008975603 | 0.889575799 |
| 30 minutes | T2-ArDP2-Bi3-Ar10keV_100-128-r340_neg54 | Roi 8 | 25679.5 | 214.6 | 0.008356861 | 0.82876022 |
| 30 minutes | T2-ArDP2-Bi3-Ar10keV_100-128-r340_neg54 | Roi 9 | 138815.1 | 965.3 | 0.006953854 | 0.690583229 |
| 30 minutes | T2-ArDP3-Bi3-Ar10keV_100-128-r340_neg58 | Roi 2 | 177275.9 | 1175.4 | 0.006630343 | 0.658667099 |
| 30 minutes | T2-ArDP3-Bi3-Ar10keV_100-128-r340_neg58 | Roi 3 | 20853.9 | 201.3 | 0.009652871 | 0.956058361 |
| 30 minutes | T2-ArDP3-Bi3-Ar10keV_100-128-r340_neg58 | Roi 4 | 10194.2 | 86.9 | 0.008524455 | 0.845240295 |
| 30 minutes | T2-ArDP3-Bi3-Ar10keV_100-128-r340_neg58 | Roi 5 | 156212 | 1262.8 | 0.008083886 | 0.801906083 |
| 30 minutes | T2-ArDP3-Bi3-Ar10keV_100-128-r340_neg58 | Roi 6 | 50396.2 | 223.4 | 0.004432874 | 0.441331026 |
| 30 minutes | T2-ArDP3-Bi3-Ar10keV_100-128-r340_neg58 | Roi 7 | 59816.3 | 475.1 | 0.007942651 | 0.78800625 |
| 30 minutes | T2-ArDP3-Bi3-Ar10keV_100-128-r340_neg58 | Roi 8 | 43470.9 | 289.2 | 0.006652726 | 0.660876003 |
| 30 minutes | T2-ArDP3-Bi3-Ar10keV_100-128-r340_neg58 | Roi 9 | 62507.9 | 472.3 | 0.007555845 | 0.749918228 |
| 30 minutes | T2-ArDP3-Bi3-Ar10keV_100-128-r340_neg58 | Roi 10 | 25304.4 | 238.4 | 0.009421286 | 0.933335421 |
| 30 minutes | T2-ArDP3-Bi3-Ar10keV_100-128-r340_neg58 | Roi 11 | 11402.1 | 90.1 | 0.007902053 | 0.784010024 |
| 30 minutes | T2-ArDP3-Bi3-Ar10keV_100-128-r340_neg58 | Roi 12 | 7787.2 | 64.1 | 0.008231457 | 0.816425305 |
| 30 minutes | T2-ArDP3-Bi3-Ar10keV_100-128-r340_neg58 | Roi 13 | 6703 | 67.1 | 0.010010443 | 0.991122731 |
| 60 minutes | T3-ArDP1-Bi3-Ar10keV_100-128-r340_neg51 | Roi 2 | 157641.8 | 2098.3 | 0.01331056 | 1.31357123 |
| 60 minutes | T3-ArDP1-Bi3-Ar10keV_100-128-r340_neg51 | Roi 3 | 62745.5 | 712.3 | 0.01135221 | 1.12247825 |
| 60 minutes | T3-ArDP1-Bi3-Ar10keV_100-128-r340_neg51 | Roi 4 | 20029.7 | 274 | 0.01367969 | 1.34950773 |
| 60 minutes | T3-ArDP1-Bi3-Ar10keV_100-128-r340_neg51 | Roi 5 | 32840.1 | 465.1 | 0.01416256 | 1.39647863 |
| 60 minutes | T3-ArDP1-Bi3-Ar10keV_100-128-r340_neg51 | Roi 6 | 7595 | 125.4 | 0.01651086 | 1.62426817 |
| 60 minutes | T3-ArDP1-Bi3-Ar10keV_100-128-r340_neg51 | Roi 2 | 22713.3 | 334 | 0.01470504 | 1.44919361 |
| 60 minutes | T3-ArDP1-Bi3-Ar10keV_100-128-r340_neg51 | Roi 3 | 18243.9 | 217 | 0.01189439 | 1.17545732 |
| 60 minutes | T3-ArDP1-Bi3-Ar10keV_100-128-r340_neg51 | Roi 4 | 42492.1 | 609 | 0.01433208 | 1.41295698 |
| 60 minutes | T3-ArDP1-Bi3-Ar10keV_100-128-r340_neg51 | Roi 5 | 503581.8 | 6654.1 | 0.01321354 | 1.30412227 |
| 60 minutes | T3-ArDP1-Bi3-Ar10keV_100-128-r340_neg51 | Roi 6 | 63384.7 | 720.6 | 0.01136867 | 1.12408802 |
| 60 minutes | T3-ArDP1-Bi3-Ar10keV_100-128-r340_neg51 | Roi 7 | 23409.6 | 343.2 | 0.01466065 | 1.44488229 |
| 60 minutes | T3-ArDP1-Bi3-Ar10keV_100-128-r340_neg51 | Roi 2 | 112943.1 | 1236 | 0.01094356 | 1.08250985 |
| 60 minutes | T3-ArDP1-Bi3-Ar10keV_100-128-r340_neg51 | Roi 3 | 40259.9 | 360.1 | 0.00894438 | 0.88650911 |
| 60 minutes | T3-ArDP1-Bi3-Ar10keV_100-128-r340_neg51 | Roi 4 | 15153.3 | 179.1 | 0.01181921 | 1.16811458 |
| 60 minutes | T3-ArDP1-Bi3-Ar10keV_100-128-r340_neg51 | Roi 5 | 149774.4 | 1954.7 | 0.01305096 | 1.28828287 |
| 60 minutes | T3-ArDP1-Bi3-Ar10keV_100-128-r340_neg51 | Roi 6 | 202948.8 | 1990.9 | 0.00980986 | 0.97145648 |
| 60 minutes | T3-ArDP1-Bi3-Ar10keV_100-128-r340_neg51 | Roi 7 | 88419.6 | 962.5 | 0.0108856 | 1.07683753 |
| 60 minutes | T3-ArDP1-Bi3-Ar10keV_100-128-r340_neg51 | Roi 8 | 394708.7 | 4696.6 | 0.0118989 | 1.17589827 |
| 120 minutes | T4-ArDP1-Bi3-Ar10keV_100-128-r340_neg30 | Roi 2 | 99983.2 | 3493.4 | 0.03493987 | 3.37602898 |
| 120 minutes | T4-ArDP1-Bi3-Ar10keV_100-128-r340_neg30 | Roi 3 | 162044.9 | 5394.9 | 0.03329262 | 3.22199382 |
| 120 minutes | T4-ArDP1-Bi3-Ar10keV_100-128-r340_neg30 | Roi 4 | 29198.8 | 799.9 | 0.02739496 | 2.66644888 |
| 120 minutes | T4-ArDP1-Bi3-Ar10keV_100-128-r340_neg30 | Roi 5 | 63167.7 | 1129.7 | 0.01788414 | 1.75699173 |
| 120 minutes | T4-ArDP1-Bi3-Ar10keV_100-128-r340_neg30 | Roi 6 | 45877.5 | 850.4 | 0.01853632 | 1.81989775 |
| 120 minutes | T4-ArDP1-Bi3-Ar10keV_100-128-r340_neg30 | Roi 7 | 59827.5 | 1451.4 | 0.02425975 | 2.3685151 |
| 120 minutes | T4-ArDP1-Bi3-Ar10keV_100-128-r340_neg30 | Roi 8 | 11322.1 | 329.9 | 0.0291377 | 2.8312736 |
| 120 minutes | T4-ArDP2-Bi3-Ar10keV_100-128-r340_neg31 | Roi 2 | 166620.5 | 7663.8 | 0.04599554 | 4.39729798 |
| 120 minutes | T4-ArDP2-Bi3-Ar10keV_100-128-r340_neg31 | Roi 3 | 43244.2 | 1475.9 | 0.03412943 | 3.30030568 |
| 120 minutes | T4-ArDP2-Bi3-Ar10keV_100-128-r340_neg31 | Roi 4 | 60519.1 | 1384.3 | 0.02287377 | 2.23622612 |
| 120 minutes | T4-ArDP2-Bi3-Ar10keV_100-128-r340_neg31 | Roi 5 | 60541 | 1233.9 | 0.02038123 | 1.99741319 |
| 120 minutes | T4-ArDP2-Bi3-Ar10keV_100-128-r340_neg31 | Roi 6 | 42521.4 | 883.2 | 0.02077072 | 2.03480737 |
| 120 minutes | T4-ArDP2-Bi3-Ar10keV_100-128-r340_neg31 | Roi 7 | 77199.8 | 3556 | 0.0460623 | 4.40339889 |
| 120 minutes | T4-ArDP2-Bi3-Ar10keV_100-128-r340_neg31 | Roi 8 | 57934.7 | 1810.6 | 0.03125243 | 3.03053127 |
| 120 minutes | T4-ArDP2-Bi3-Ar10keV_100-128-r340_neg31 | Roi 9 | 16018.3 | 581.7 | 0.03631472 | 3.50421687 |
| 120 minutes | T4-ArDP2-Bi3-Ar10keV_100-128-r340_neg31 | Roi 10 | 12759.5 | 514.6 | 0.04033073 | 3.87672234 |
| 120 minutes | T4-ArDP2-Bi3-Ar10keV_100-128-r340_neg31 | Roi 11 | 11784.4 | 145.3 | 0.01232986 | 1.2179686 |
| 120 minutes | T4-ArDP2-Bi3-Ar10keV_100-128-r340_neg31 | Roi 12 | 26716.7 | 696.3 | 0.02606235 | 2.54003575 |
| 120 minutes | T4-ArDP2-Bi3-Ar10keV_100-128-r340_neg31 | Roi 13 | 10414.3 | 427.6 | 0.04105893 | 3.94395816 |
| 240 minutes | T5-ArDP1-Bi3-Ar10keV_100-128-r340_neg17 | Roi 2 | 44279.3 | 3271.2 | 0.07387651 | 6.87942293 |
| 240 minutes | T5-ArDP1-Bi3-Ar10keV_100-128-r340_neg17 | Roi 3 | 34852.6 | 2663.1 | 0.07641037 | 7.09862804 |
| 240 minutes | T5-ArDP1-Bi3-Ar10keV_100-128-r340_neg17 | Roi 4 | 25921.4 | 1641.7 | 0.06333377 | 5.95615152 |
| 240 minutes | T5-ArDP1-Bi3-Ar10keV_100-128-r340_neg17 | Roi 5 | 33204.8 | 1514.8 | 0.04561991 | 4.36295349 |
| 240 minutes | T5-ArDP1-Bi3-Ar10keV_100-128-r340_neg17 | Roi 6 | 6485.4 | 111.7 | 0.0172233 | 1.69316821 |
| 240 minutes | T5-ArDP1-Bi3-Ar10keV_100-128-r340_neg17 | Roi 7 | 48020.8 | 2796.3 | 0.05823102 | 5.50267528 |
| 240 minutes | T5-ArDP1-Bi3-Ar10keV_100-128-r340_neg17 | Roi 8 | 22276.8 | 1571.1 | 0.07052629 | 6.58800146 |
| 240 minutes | T5-ArDP2-Bi3-Ar10keV_100-128-r340_neg22 | Roi 2 | 51437.8 | 3934.3 | 0.07648655 | 7.1052028 |
| 240 minutes | T5-ArDP2-Bi3-Ar10keV_100-128-r340_neg22 | Roi 3 | 145964.4 | 10604 | 0.07264785 | 6.77275874 |
| 240 minutes | T5-ArDP2-Bi3-Ar10keV_100-128-r340_neg22 | Roi 4 | 80484.2 | 6999.2 | 0.08696365 | 8.00060354 |
| 240 minutes | T5-ArDP2-Bi3-Ar10keV_100-128-r340_neg22 | Roi 5 | 73791.7 | 6039.7 | 0.08184796 | 7.56556944 |
| 240 minutes | T5-ArDP2-Bi3-Ar10keV_100-128-r340_neg22 | Roi 6 | 31936.8 | 2211.1 | 0.06923361 | 6.47506875 |
| 240 minutes | T5-ArDP2-Bi3-Ar10keV_100-128-r340_neg22 | Roi 7 | 27442.7 | 2255.6 | 0.08219308 | 7.59504753 |
| 240 minutes | T5-ArDP2-Bi3-Ar10keV_100-128-r340_neg22 | Roi 8 | 26581.1 | 1872.8 | 0.07045608 | 6.58187454 |
| 240 minutes | T5-ArDP2-Bi3-Ar10keV_100-128-r340_neg22 | Roi 9 | 7369.4 | 394.6 | 0.05354574 | 5.08243174 |
| 240 minutes | T5-ArDP3-Bi3-Ar10keV_100-128-r340_neg23 | Roi 2 | 18638.6 | 298 | 0.01598833 | 1.57367215 |
| 240 minutes | T5-ArDP3-Bi3-Ar10keV_100-128-r340_neg23 | Roi 3 | 21450.7 | 1308.3 | 0.06099102 | 5.7484951 |
| 240 minutes | T5-ArDP3-Bi3-Ar10keV_100-128-r340_neg23 | Roi 4 | 56143 | 1847.5 | 0.03290704 | 3.18586665 |
| 240 minutes | T5-ArDP3-Bi3-Ar10keV_100-128-r340_neg23 | Roi 5 | 32273 | 2462.3 | 0.07629597 | 7.08875409 |
| 240 minutes | T5-ArDP3-Bi3-Ar10keV_100-128-r340_neg23 | Roi 6 | 78621.6 | 6018.8 | 0.07655403 | 7.111025 |
| 360 minutes | T6-ArDP1-Bi3-Ar10keV_100-128-r340_0109neg10 | Roi 2 | 6980.5 | 731.1 | 0.10473462 | 9.48052285 |
| 360 minutes | T6-ArDP1-Bi3-Ar10keV_100-128-r340_0109neg10 | Roi 3 | 13070.3 | 1920.1 | 0.14690558 | 12.8088643 |
| 360 minutes | T6-ArDP1-Bi3-Ar10keV_100-128-r340_0109neg10 | Roi 4 | 13850 | 1880.7 | 0.13579061 | 11.9556027 |
| 360 minutes | T6-ArDP1-Bi3-Ar10keV_100-128-r340_0109neg10 | Roi 5 | 6369.3 | 1128 | 0.17709952 | 15.0454163 |
| 360 minutes | T6-ArDP1-Bi3-Ar10keV_100-128-r340_0109neg10 | Roi 6 | 57964.6 | 6940.3 | 0.11973342 | 10.6930293 |
| 360 minutes | T6-ArDP1-Bi3-Ar10keV_100-128-r340_0109neg10 | Roi 7 | 17116 | 2268.4 | 0.13253097 | 11.7021935 |
| 360 minutes | T6-ArDP1-Bi3-Ar10keV_100-128-r340_0109neg10 | Roi 8 | 4068.1 | 798.8 | 0.19635702 | 16.4129117 |
| 360 minutes | T6-ArDP1-Bi3-Ar10keV_100-128-r340_0109neg10 | Roi 9 | 5135.4 | 983.2 | 0.19145539 | 16.0690354 |
| 360 minutes | T6-ArDP1-Bi3-Ar10keV_100-128-r340_0109neg10 | Roi 10 | 3632.1 | 344.1 | 0.09473858 | 8.65399125 |
| 360 minutes | T6-ArDP2-Bi3-Ar10keV_100-128-r310_neg9 | Roi 1 | 42298.2 | 6054.4 | 0.14313611 | 12.5213536 |
| 360 minutes | T6-ArDP2-Bi3-Ar10keV_100-128-r310_neg9 | Roi 2 | 12420.1 | 1362 | 0.10966095 | 9.88238367 |
| 360 minutes | T6-ArDP2-Bi3-Ar10keV_100-128-r310_neg9 | Roi 3 | 5049.3 | 686.5 | 0.13595944 | 11.9686879 |
| 360 minutes | T6-ArDP2-Bi3-Ar10keV_100-128-r310_neg9 | Roi 4 | 6213.7 | 797.5 | 0.12834543 | 11.3746577 |
| 360 minutes | T6-ArDP2-Bi3-Ar10keV_100-128-r310_neg9 | Roi 5 | 5508.4 | 978.9 | 0.17771041 | 15.0894825 |
| 360 minutes | T6-ArDP2-Bi3-Ar10keV_100-128-r310_neg9 | Roi 6 | 17353.4 | 2408.7 | 0.13880277 | 12.188482 |
| 360 minutes | T6-ArDP2-Bi3-Ar10keV_100-128-r310_neg9 | Roi 7 | 5145.8 | 1164.9 | 0.22637879 | 18.459125 |
| 360 minutes | T6-ArDP2-Bi3-Ar10keV_100-128-r310_neg9 | Roi 8 | 1987 | 470.7 | 0.23688978 | 19.1520527 |
| 360 minutes | T6-ArDP2-Bi3-Ar10keV_100-128-r310_neg9 | Roi 9 | 3980 | 401.6 | 0.10090452 | 9.16560161 |
| 360 minutes | T6-ArDP2-Bi3-Ar10keV_100-128-r310_neg9 | Roi 10 | 2716.5 | 388.6 | 0.14305172 | 12.5148949 |
| 360 minutes | T6-ArDP2-Bi3-Ar10keV_100-128-r310_neg9 | Roi 11 | 1776.9 | 242 | 0.13619224 | 11.9867254 |
| 360 minutes | T6-ArDP2-Bi3-Ar10keV_100-128-r310_neg9 | Roi 12 | 2494.5 | 84.3 | 0.03379435 | 3.26896231 |
| 360 minutes | T6-ArDP2-Bi3-Ar10keV_100-128-r310_neg9 | Roi 13 | 2262.4 | 111.8 | 0.04941655 | 4.7089546 |
| 360 minutes | T6-ArDP2-Bi3-Ar10keV_100-128-r310_neg9 | Roi 14 | 1351.8 | 114.9 | 0.08499778 | 7.83391287 |
| 360 minutes | T6-ArDP2-Bi3-Ar10keV_100-128-r310_neg9 | Roi 15 | 2101 | 108.6 | 0.05168967 | 4.91491673 |
| 360 minutes | T6-ArDP2-Bi3-Ar10keV_100-128-r310_neg9 | Roi 16 | 2765.5 | 276 | 0.09980112 | 9.07446983 |
| 360 minutes | T6-ArDP2-Bi3-Ar10keV_100-128-r310_neg9 | Roi 17 | 7006.6 | 1358.7 | 0.19391716 | 16.2420953 |
| 360 minutes | T6-ArDP2-Bi3-Ar10keV_100-128-r310_neg9 | Roi 18 | 34189.6 | 4527.6 | 0.13242623 | 11.6940275 |
| 360 minutes | T6-ArDP3-Bi3-Ar10keV_100-128-r310_neg10 | Roi 1 | 80001.3 | 11993.6 | 0.14991756 | 13.0372445 |
| 360 minutes | T6-ArDP3-Bi3-Ar10keV_100-128-r310_neg10 | Roi 2 | 59042.3 | 8742.3 | 0.14806842 | 12.8971772 |
| 360 minutes | T6-ArDP3-Bi3-Ar10keV_100-128-r310_neg10 | Roi 3 | 3146 | 829.5 | 0.26366815 | 20.8653 |
| 360 minutes | T6-ArDP3-Bi3-Ar10keV_100-128-r310_neg10 | Roi 4 | 15633.5 | 1969 | 0.12594748 | 11.1859111 |
| 360 minutes | T6-ArDP3-Bi3-Ar10keV_100-128-r310_neg10 | Roi 5 | 1853 | 237.5 | 0.12817053 | 11.3609184 |
| 360 minutes | T6-ArDP3-Bi3-Ar10keV_100-128-r310_neg10 | Roi 6 | 14509.6 | 2719.4 | 0.18742074 | 15.7838528 |
| 360 minutes | T6-ArDP3-Bi3-Ar10keV_100-128-r310_neg10 | Roi 7 | 4872.6 | 240.6 | 0.04937816 | 4.7054682 |
| 360 minutes | T6-ArDP3-Bi3-Ar10keV_100-128-r310_neg10 | Roi 8 | 7690.6 | 1751.7 | 0.22777157 | 18.5516241 |
| 360 minutes | T6-ArDP3-Bi3-Ar10keV_100-128-r310_neg10 | Roi 9 | 3258.3 | 320.3 | 0.0983028 | 8.95042754 |
| 360 minutes | T6-ArDP3-Bi3-Ar10keV_100-128-r310_neg10 | Roi 10 | 4344.1 | 693.2 | 0.15957275 | 13.7613404 |
| 360 minutes | T6-ArDP3-Bi3-Ar10keV_100-128-r310_neg10 | Roi 12 | 1953.7 | 11 | 0.00563034 | 0.55988192 |
| 360 minutes | T6-ArDP3-Bi3-Ar10keV_100-128-r310_neg10 | Roi 13 | 1629 | 118.2 | 0.07255985 | 6.76510989 |
| 360 minutes | T6-ArDP6-Bi3-Ar10keV_100-128-r340_neg14 | Roi 2 | 4138.9 | 631.4 | 0.15255261 | 13.2360648 |
| 360 minutes | T6-ArDP6-Bi3-Ar10keV_100-128-r340_neg14 | Roi 3 | 3462.2 | 588.3 | 0.16992086 | 14.5241328 |
| 360 minutes | T6-ArDP6-Bi3-Ar10keV_100-128-r340_neg14 | Roi 4 | 6158.8 | 912.7 | 0.14819445 | 12.9067383 |
| 360 minutes | T6-ArDP6-Bi3-Ar10keV_100-128-r340_neg14 | Roi 5 | 11142.2 | 1389.9 | 0.12474197 | 11.090719 |
| 360 minutes | T6-ArDP6-Bi3-Ar10keV_100-128-r340_neg14 | Roi 6 | 5432.5 | 582.5 | 0.10722503 | 9.68412303 |
| 360 minutes | T6-ArDP6-Bi3-Ar10keV_100-128-r340_neg14 | Roi 7 | 2189.1 | 220.4 | 0.10068065 | 9.14712596 |
| 360 minutes | T6-ArDP6-Bi3-Ar10keV_100-128-r340_neg14 | Roi 8 | 5289.8 | 360.8 | 0.06820674 | 6.38516264 |
| 360 minutes | T6-ArDP6-Bi3-Ar10keV_100-128-r340_neg14 | Roi 9 | 2282.3 | 375.1 | 0.16435175 | 14.1153007 |
| 360 minutes | T6-ArDP6-Bi3-Ar10keV_100-128-r340_neg14 | Roi 10 | 1906.4 | 207.8 | 0.10900126 | 9.82877684 |
| 360 minutes | T6-ArDP6-Bi3-Ar10keV_100-128-r340_neg14 | Roi 11 | 1602.3 | 108.2 | 0.06752793 | 6.32563578 |
| 360 minutes | T6-ArDP6-Bi3-Ar10keV_100-128-r340_neg14 | Roi 12 | 1365.5 | 335.6 | 0.24577078 | 19.728411 |
| 360 minutes | T6-ArDP6-Bi3-Ar10keV_100-128-r340_neg14 | Roi 13 | 1640.4 | 222.4 | 0.13557669 | 11.9390165 |
| 360 minutes | T6-ArDP6-Bi3-Ar10keV_100-128-r340_neg14 | Roi 14 | 2399.4 | 309.7 | 0.12907394 | 11.4318408 |
